# Sequential targeting of interferon pathways for increased host resistance to bacterial superinfection during influenza

**DOI:** 10.1371/journal.ppat.1009405

**Published:** 2021-03-09

**Authors:** Tarani Kanta Barman, Rachael Racine, Jesse L. Bonin, Danielle Califano, Sharon L. Salmon, Dennis W. Metzger

**Affiliations:** Department of Immunology and Microbial Disease, Albany Medical College, Albany, New York, United States of America; University of Geneva, SWITZERLAND

## Abstract

Bacterial co-infections represent a major clinical complication of influenza. Host-derived interferon (IFN) increases susceptibility to bacterial infections following influenza, but the relative roles of type-I versus type-II IFN remain poorly understood. We have used novel mouse models of co-infection in which colonizing pneumococci were inoculated into the upper respiratory tract; subsequent sublethal influenza virus infection caused the bacteria to enter the lungs and mediate lethal disease. Compared to wild-type mice or mice deficient in only one pathway, mice lacking both IFN pathways demonstrated the least amount of lung tissue damage and mortality following pneumococcal-influenza virus superinfection. Therapeutic neutralization of both type-I and type-II IFN pathways similarly provided optimal protection to co-infected wild-type mice. The most effective treatment regimen was staggered neutralization of the type-I IFN pathway early during co-infection combined with later neutralization of type-II IFN, which was consistent with the expression and reported activities of these IFNs during superinfection. These results are the first to directly compare the activities of type-I and type-II IFN during superinfection and provide new insights into potential host-directed targets for treatment of secondary bacterial infections during influenza.

## Author summary

Bacterial co-infections represent a common and challenging clinical complication of influenza. Type-I and type-II interferon (IFN) pathways enhance susceptibility to influenza-pneumococcal co-infection, leading to increased lung pathology and mortality. However, the comparative importance of type-I versus type-II IFN remains unclear. We have used two novel mouse models of co-infection in which pneumococci were inoculated into the upper respiratory tract followed two days later by influenza virus infection. Virus co-infection caused IFN-dependent inflammation that facilitated spreading of the colonizing bacteria into the lungs, followed by tissue damage and death. In this pneumococcal-influenza virus superinfection model, mice lacking both type-I and type-II IFN pathways demonstrated minimal lung pathology and increased survival compared to wild-type mice and mice deficient in only one pathway. Therapeutic neutralization of both type-I and type-II IFN pathways similarly provided optimal protection to superinfected wild-type mice. The most effective treatment regimen involved neutralization of the type-I IFN pathway early during co-infection combined with later neutralization of the type-II IFN pathway. These results provide new insights into potential host-directed therapy for management of bacterial-viral superinfections.

## Introduction

Influenza A virus is a leading cause of respiratory infection in the United States. Complications involving secondary infections with bacterial pathogens, such as *Streptococcus pneumoniae*, significantly exacerbate the risk of severe disease and result in considerably increased rates of hospitalization and death [1]. It is estimated that at least 95% of the deaths that occurred during the 1918 pandemic were due to influenza-associated pneumococcal lung infection [2]. Similarly, approximately half of hospitalized patients during the 1957 and 2009 influenza pandemics presented with bacterial co-infections [3,4]. Influenza can promote bacterial pneumonia through epithelial damage, inflammation in the respiratory tract, and suppression of innate lung immune responses [5,6]. Evidence from human and mouse studies indicates that influenza infection compromises both the host immune response and lung barrier function to promote increased susceptibility to bacterial superinfection.

There is now general consensus in the field that host cytokine responses during influenza are crucial in mediating susceptibility to secondary bacterial infection. However, the precise roles of individual cytokines remain unclear. In particular, multiple groups have reported that virus-induced type-I interferon (IFN) results in increased susceptibility to secondary bacterial infection [7–11]. Others, including our own group, have instead defined a critical role for type-II IFN in mediating superinfection during influenza [12–16]. The relative importance of type-I versus type-II IFN has thus remained confusing, especially since in every case, neutralization of either cytokine alone has been found to partially protect from death. The design of co-infection experiments in the majority of mouse studies–infection with influenza virus followed by pulmonary challenge with bacteria-may be in part responsible for this uncertainty. In humans, it is believed that superinfection results from bacteria colonizing the upper respiratory tract, which are then aspirated into the lungs during subsequent influenza. Weiser’s group has shown that influenza-mediated inflammation in the upper respiratory tract of mice can increase pneumococcal colonization, which then facilitates microaspiration, and that type-I IFN is critically responsible for this effect [8,17]. Mouse studies in which bacteria are directly inoculated into the lungs of mice following influenza actually reverse the timing of human co-infection and do not accurately mimic the clinical scenario. Thus, a colonization model of pneumococcus followed by influenza A virus infection is more relevant to human co-infection than traditional murine experiments in which influenza virus is inoculated before pneumococcus infection. Another possible confounding factor is that type-I IFN is known to increase host resistance to virulent respiratory viruses. Thus, neutralization of type-I IFN could exacerbate viral infection which in turn, might lead to increased susceptibility to co-infection. Neutralization of type-II IFN, on the other hand, either has no effect on viral disease or increases ILC2-mediated lung tissue healing [18,19].

In the current study, we have now directly addressed the relative importance of type-I and type-II IFNs in mediating susceptibility to pneumococcal infection during influenza. We used experimental models in which mice were inoculated with pneumococci before influenza virus challenge, in order to better model human colonization and co-infection. Our results show that in fact, both type-I and type-II IFNs play complementary and essential roles in mediating susceptibility to pneumococcal infection during influenza. Type-I IFN is most critical during early bacterial infection of the upper respiratory tract while type-II IFN inhibits bacterial clearance from the lower respiratory tract at later stages of infection. These findings resolve a major outstanding issue in the field and suggest new therapeutic approaches for prevention of lethal bacterial superinfections in humans.

## Results

### Pneumococcal-influenza co-infection model

**Fig 1:**
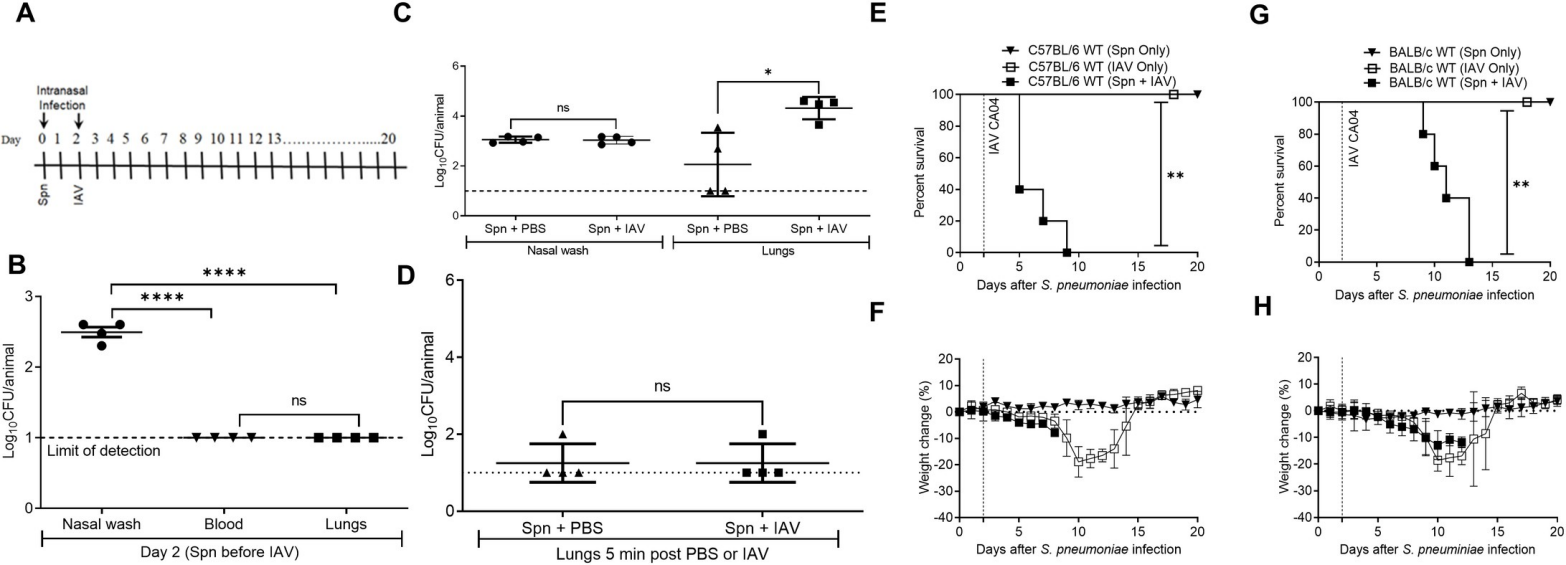
Superinfection with sublethal *S*. *pneumoniae* followed by influenza virus causes mortality in C57BL/6 and BALB/c mice. **(A)** Experimental protocol for superinfection**. (B)** Bacterial burdens in nasal washes, blood and lungs 48 h after pneumococcal infection and before viral co-infection. Each symbol represents the CFU of an individual mouse with the solid lines showing mean ± SD from 4 mice/group. The dotted line indicates the limit of detection. **(C)** Nasal wash and lung bacterial burdens on Day 4 following Day 0 intranasal inoculation of 20 μL PBS containing 10^3^ CFU of *S*. *pneumoniae* (Spn) followed by Day 2 inoculation of 40 μL PBS alone or PBS containing 50 PFU of influenza A virus (IAV). **(D)** Lung bacterial burdens after intranasal inoculation of 20 μL PBS containing 10^3^ CFU of Spn on Day 0 and a 5 min inoculation of 40 μL PBS alone or PBS containing 50 PFU of IAV on Day 2. (**E-G**) Morbidity and mortality of Spn-IAV super-infected C57BL/6 mice and BALB/c mice. (**E**) Survival and (**F**) weight loss of C57BL/6 mice (n = 5 mice/group). (**G**) Survival and (**H**) weight loss of BALB/c mice (n = 5 mice/group). Statistical analyses for **(B-D)** were performed by two-way ANOVA and survival data were analyzed by the log-rank Mantel-Cox test. *P<0.05; **P<0.01; ****P<0.0001; ns = not significant.

Nasopharyngeal carriage of *S*. *pneumoniae* in humans often leads to pneumococcal disease [20]. To model pneumococcal carriage limited to the upper respiratory tract, we inoculated lightly anesthetized mice with a low volume of A66.1 *S*. *pneumoniae* (Fig 1A). After 48 h, the mice were evaluated for bacterial loads in nasal washes, blood, and lungs. Bacteria were detected at this time point in nasal washes at levels approximating the initial inoculum dose, while no bacteria were observed in the bloodstream or in lung homogenates (Fig 1B). We next tested the effects of influenza co-infection following pneumococcal inoculation. For this purpose, a sublethal dose of CA04 virus, the H1N1 strain responsible for the 2009 pandemic and associated with a high degree of bacterial co-infection, was intranasally administered to anesthetized mice on Day 2 after pneumococcal infection (Fig 1A). In preliminary experiments to establish synergistic superinfection conditions, we tested 3 different doses of pneumococci (10^2^, 10^3^ and 10^4^ CFU) and 3 different doses of influenza virus (10, 50 and 100 PFU), and chose the doses that caused the least amount of mortality in singly-infected mice and the most reproducible synergy in co-infected mice. Out of these various inoculum doses, 10^3^ CFU *S*. *pneumoniae* and 50 PFU of CA04 were chosen for use in the experiments.

After infection with pneumococci alone, low levels of bacteria were detected in the lungs of about half of the animals on Day 4, but after viral superinfection (2 days after viral infection) all mice contained significantly elevated levels of bacteria in the lungs (Fig 1C). Presence of bacteria in the lungs after co-infection was not due solely to washing of the bacteria from the upper respiratory tract into the lung during the virus inoculation procedure as lungs collected 5 minutes after either viral or PBS inoculation failed to contain significant numbers of bacterial CFU (Fig 1D). In addition, increased lung bacterial outgrowth occurred using even 4-fold lower volumes of PBS for virus infection (S1 Fig). H1N1 CA04 virus infection induced a significant level of inflammation in the upper respiratory tract as shown by an influx of neutrophils (S2 Fig). Compared to mice infected with *S*. *pneumoniae* alone, the number of bacteria in the nasal washes of co-infected mice increased and then remained consistent through Day 8 (S3 Fig). These results confirm the reports of others [8], who concluded that induced inflammation increased the ability of *S*. *pneumoniae* to colonize and spread into the lower respiratory tract.

We next examined morbidity and mortality in pneumococcal-influenza superinfected mice. In mice inoculated intranasally with either 10^3^ CFU of pneumococci or 50 PFU of influenza virus alone, no mortality was observed (Fig 1E and 1G). Weight loss was seen following viral infection as expected [18] but not with pneumococcal infection (Fig 1F and 1H). Upon co-infection of C57BL/6 mice, all animals succumbed between Days 5–9 (Fig 1E). The same result was obtained with BALB/c mice, although time to death was somewhat delayed and occurred between Days 9 and 13 (Fig 1G).

**Fig 2:**
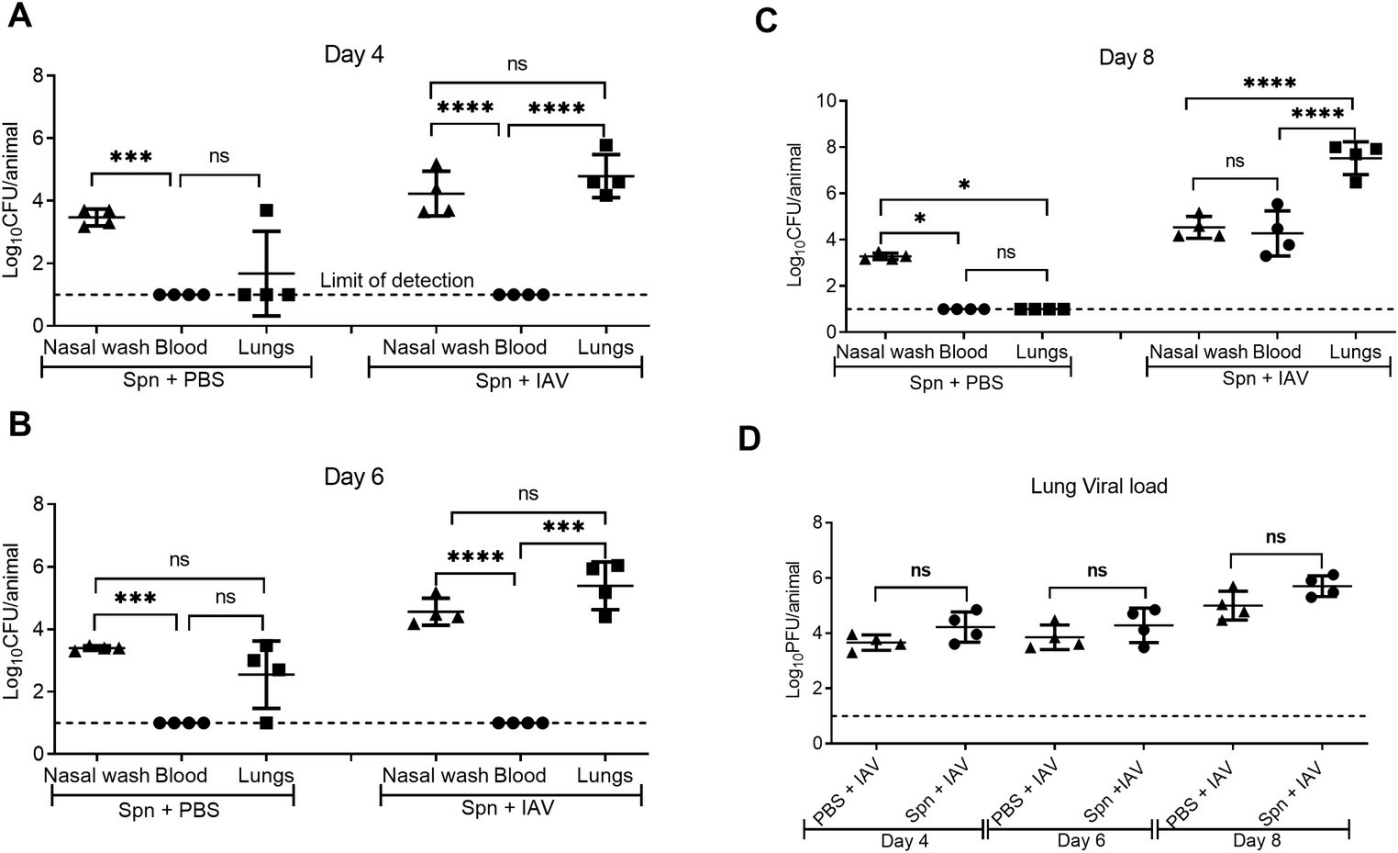
Pathogen loads in pneumococcal-influenza virus superinfected mice. Bacterial burdens in nasal washes, blood and lung tissues of BALB/c mice on (**A**) Day 4, (**B**) Day 6, and (**C**) Day 8 after Day 0 Spn infection followed by inoculation of PBS or IAV on Day 2. Each symbol represents the CFU of an individual mouse with the solid lines showing mean ± SD from 4 mice/group. The dotted line indicates the limit of detection. (**D**) Viral burdens in the lung tissues of mice co-infected with Spn on Day 0 and IAV on Day 2 or IAV alone on Day 2. Each symbol represents the PFU of an individual mouse with the solid lines showing mean ± SD from 4 mice/group. The dotted line indicates the limit of detection. Statistical analyses were performed by two-way ANOVA. *P<0.05; ***P<0.001; ****P<0.0001; ns = not significant.

A correlation was observed between the kinetics of bacterial outgrowth and decreased survival in BALB/c mice following H1N1 CA04 virus co-infection. Bacterial counts in nasal washes remained consistent from Days 4 to 8, confirming nasopharyngeal carriage (Figs 2A–2C and S3). Lung bacterial counts increased from 4.79 log_10_ ± 0.69 log_10_ on Day 4 to 7.53 log_10_ ± 0.71 log_10_ on Day 8, levels that were significantly greater than those seen in nasal washes. Bacteria in the blood remained undetectable on Days 4 and 6, but were detected on Day 8, shortly before the mice succumbed. In mice infected with only *S*. *pneumoniae*, relative bacterial counts in the lungs were insignificant and the bacteria were cleared by Day 8. Pneumococcal-influenza co-infection did not alter viral burden compared to mice infected with influenza virus alone (Fig 2D).

### Cell and cytokine expression

**Fig 3:**
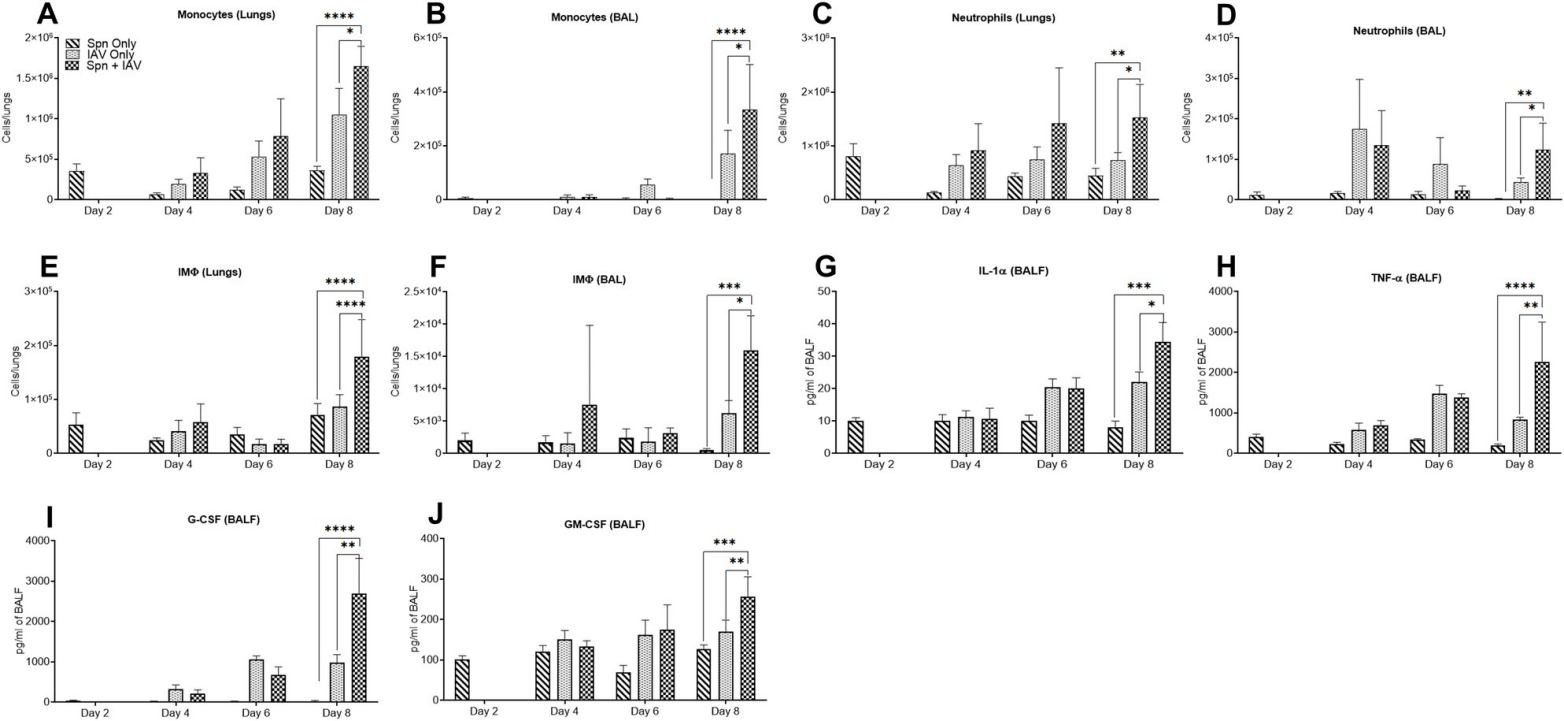
Cell and cytokine profiles in the lungs of pneumococcal-influenza virus superinfected mice. BALB/c mice were infected with Spn on Day 0 and cell subsets in the lungs and BAL were analyzed on Days 2, 4, 6 and 8. In the second group, mice were treated with PBS on Day 0 and infected with IAV on Day 2 and the lungs and BAL were analyzed on Days 4, 6 and 8. The third group was infected with Spn on Day 0 and IAV on Day 2, and then analyzed on Days 4, 6, and 8. Numbers of (**A-B**) monocytes, (**C-D**) neutrophils and (**E-F**) interstitial macrophages were evaluated by flow cytometry. Levels of (**G**) IL-1α, (**H**) TNF-α, (**I**) G-CSF, and (**J**) GM-CSF were evaluated in BALF by Luminex assay. Data are presented as mean ± SD from 4 mice/group. Statistical analyses were performed by two-way ANOVA. *P<0.05; **P<0.01; ***P<0.001; ****P<0.0001.

In addition to measuring bacterial and viral lung burdens, we examined expression of various immune cell subsets in lung tissues and bronchoalveolar lavage (BAL) of BALB/c mice. Of the various cells examined (gating strategies shown in S4 Fig), monocytes, neutrophils, and interstitial macrophages were found to be expressed at significantly greater levels in pneumococcal-influenza co-infected mice compared to mice infected with either pneumococcus or influenza virus alone, again primarily at a time point just before the mice succumbed to infection (Fig 3A–3F). There were no significant differences in expression of eosinophils, alveolar macrophages, or T cells between co-infected versus singly-infected mice that could account for increased mortality during co-infection. IL-1α, TNF-α, G-CSF and GM-CSF levels were also found to be significantly greater in co-infected mice compared to mice infected with either pathogen alone (Fig 3G–3J). These results suggest greater infiltration of inflammatory cells into the lungs of superinfected mice, together with increased production of inflammatory cytokines.

### Deficiency in type-I and type-II IFN decreases susceptibility to co-infection

**Fig 4:**
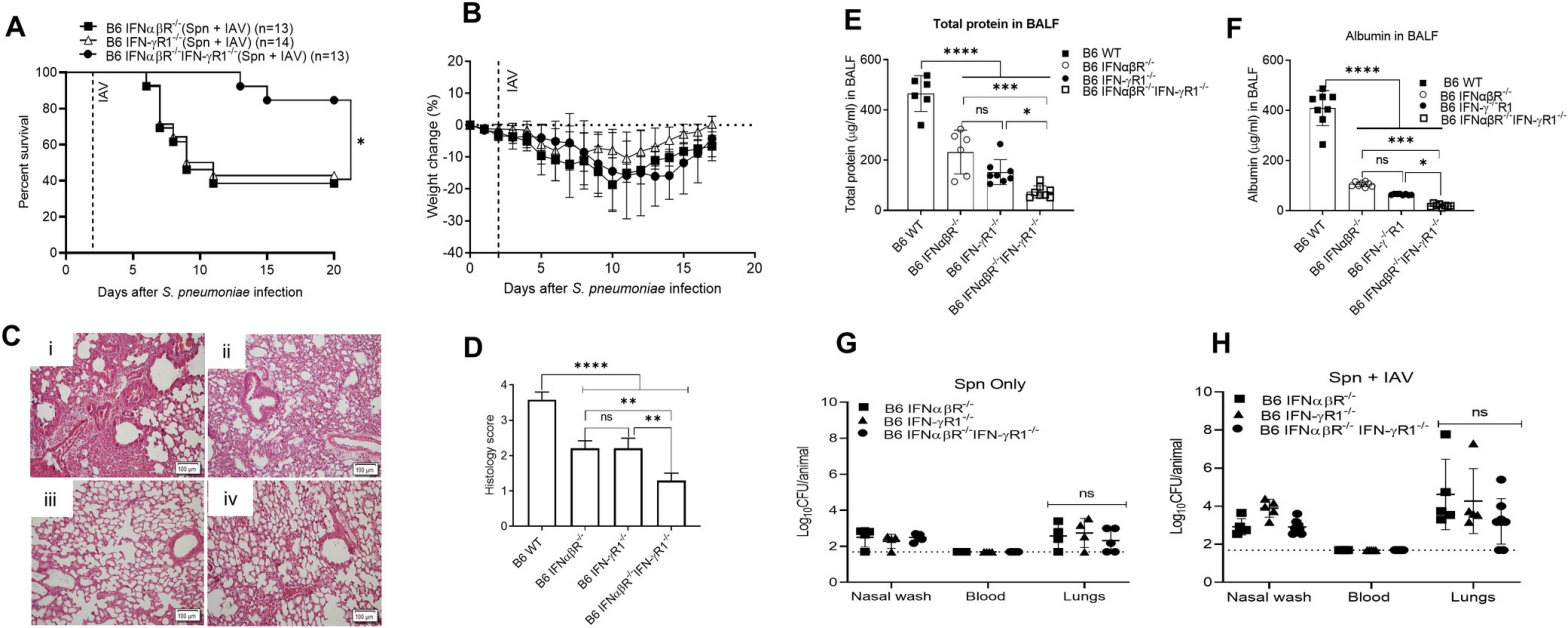
Susceptibility of mice deficient in type-I and/or type-II IFN pathways to pneumococcal-influenza virus superinfection. **(A,B)**, C57BL/6 IFNαβR^-/-^ (n = 13), IFN-γR1^-/-^ (n = 14) and IFNαβR^-/-^IFN-γR1^-/-^ (n = 13) mice were monitored for (**A)** survival and (**B**) weight loss following intranasal infection with 10^3^ Spn on Day 0 and 50 PFU of IAV on Day 2. Survival data were analyzed by log-rank Mantel-Cox test. (**C)** Histopathology on Day 7 of co-infection. (**Ci**) wild type, (**Cii**) IFN-γR1^-/-^, (**Ciii**) IFNαβR^-/-^ and (**Civ**) IFNαβR^-/-^ IFN-γR1^-/-^ mice. 20X magnification; scale = 100μm. (**D**) Histology scores for same groups shown in **(C**). The lesions were scored for levels of inflammatory infiltrates, edema, hyperemia and congestion, alveolar degeneration, necrosis of alveolar epithelium, necrotizing bronchitis and bronchiolitis. The scoring criteria were 0 for no change, 1 for mild change, 2 for moderate changes, 3 for marked changes and 4 for severe changes. 10 random fields per mouse were evaluated. Shown are mean ± SD for 3–5 mice/group. BALF were analyzed for biomarkers of tissue integrity (**E)** total protein, and **(F**) albumin. Each symbol represents an individual mice showing mean ± SD from 6 to 8 mice/group. (**G,H)** Bacterial loads in nasal washes, blood and lungs of (**G)** Spn only or **(H**) Spn-IAV co-infected C57BL/6 IFNαβR^-/-^, IFN-γR1^-/-^ and IFNαβR^-/-^IFN-γR1^-/-^ mice. Each symbol represents the CFU of an individual mouse with the solid lines showing mean ± SD from 4–8 mice. The dotted line indicates the limit of detection. Statistical analyses for (**D-H**) were performed by two-way ANOVA. *P<0.05; **P<0.01, ****P<0.0001; ns = not significant. The data in (**A,B,G, and H**) were pooled from two independent experiments.

Mice deficient in either the type-I or type-II IFN pathway show increased survival from secondary pneumococcal infection following influenza compared to WT mice [8,9,15,16]. We therefore tested whether such animals would also demonstrate heightened resistance in our model of bacterial infection followed by viral challenge. C57BL/6 IFNαβR^-/-^ and IFN-γR1^-/-^ mice were inoculated with 10^3^ CFU of *S*. *pneumoniae* A66.1 on Day 0 and 50 PFU of H1N1 CA04 virus on Day 2, followed by daily monitoring for weight loss and survival. While WT mice all succumbed to co-infection (Fig 1), each KO strain demonstrated approximately 40% survival (Fig 4A and 4B). Similar partial resistance to co-infection was observed utilizing BALB/c IFNαβR^-/-^ mice (S5A and S5B Fig) as well as C57BL/6 and BALB/c IFN-γ^-/-^ mice (S6A–S6D Fig). However, use of mice lacking both cytokine pathways (C57BL/6 IFNαβR^-/-^ IFN-γR1^-/-^ double KO mice) revealed significantly increased resistance such that nearly all of these animals survived superinfection (Fig 4A and 4B). KO mice infected with either pathogen alone all survived (S5A and S5B and S6A–S6D Fig). Absence of type-I IFN signaling did not affect expression of type-II IFN in this model. These data indicate that both type-I and type-II IFNs play critical and possibly complementary roles in mediating susceptibility in the bacterial-viral co-infection model. In addition to mortality, we performed histopathology analysis of Day 7 lung tissues. The IFNαβR^-/-^ IFN-γR1^-/-^ double KO mice exhibited significantly less tissue pathology compared to WT mice after co-infection (Fig 4C, panels i versus iv). Mice lacking just one IFN pathway (IFNαβR^-/-^ or IFN-γR1^-/-^ mice) (Fig 4C, panels ii and iii) also showed significant differences from WT mice, indicating that both cytokines play roles in loss of tissue integrity during co-infection, yet mice deficient in both cytokine pathways displayed the least amount of damage (Fig 4D). We also measured total protein (Fig 4E) and albumin (Fig 4F) in BALF as indicators of barrier integrity and the results confirmed the histology analysis. Evaluation of Day 7 bacterial burdens showed low levels of pneumococci in both nasal washes and lung homogenates of all KO mice (Fig 4G and 4H). Increases in nasal wash bacterial levels were seen only in the presence of type I IFN signaling (only in IFN-γR1^-/-^ mice but not IFNαβR^-/-^ mice nor IFNαβR^-/-^ IFN-γ^-/-^ mice) while significant increases in lung bacterial numbers were observed only in the presence of the type II IFN signaling pathway (only in IFNαβR^-/-^ mice) (S7 Fig). Thus, increased colonization was dependent upon the presence of type I IFN signaling, in agreement with the results of others [8]. Notably, no bacteria were detectable in the blood of any of the mice.

### Staggered neutralization of both type-I and type-II IFN rescues wild-type mice

**Fig 5:**
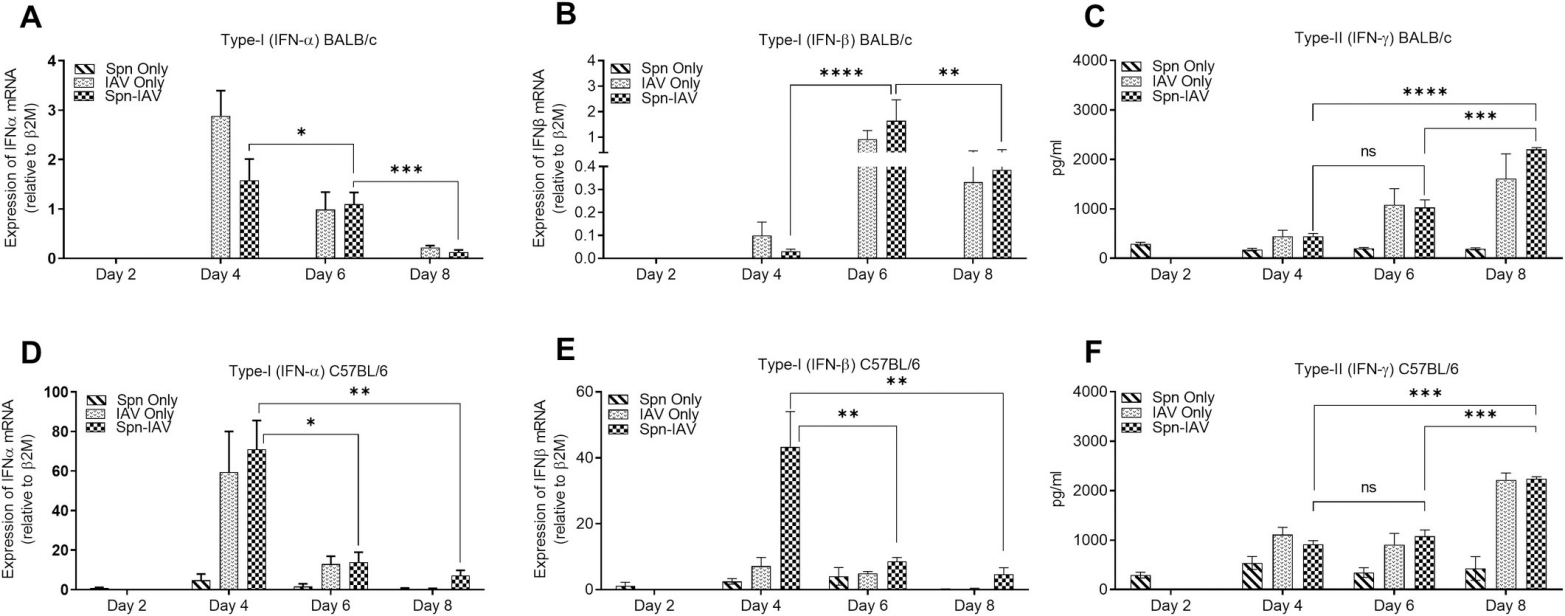
Expression of type-I and type-II IFNs in pneumococcal-influenza virus superinfected mice. BALB/c and C57BL/6 mice were infected with *S*. *pneumoniae* on Day 0 and IAV H1N1 CA04 on Day 2 and whole lung tissue samples were collected on Days 2, 4, 6 and 8. Day 2 sampling was performed before IAV infection. Quantification of type-I IFNs was performed by RT-qPCR and type-II IFN by IFN-γ ELISA. Expression of IFN-α mRNA in BALB/c (**A)** and C57BL/6 (**D**) lung tissues on Days 2, 4, 6 and 8. Expression of IFN-β mRNA in BALB/c (**B)** and C57BL/6 (**E**) lung tissues on Days 2, 4, 6 and 8. Expression of IFN-γ in BALB/c (**C)** and C57BL/6 (**F**) BALF on Days 2, 4, 6, and 8. Data are presented as means ± SD from 4 mice/group. Statistical analyses were performed by two-way ANOVA. *P*>0.05; *P<0.05; **P<0.01, ***P<0.001.

Based on the above results, we measured expression of IFN-α, IFN-β and IFN-γ in lungs of BALB/c and C57BL/6 WT mice on Days 2–8 following pneumococcal-influenza co-infection (Fig 5A–5F). IFN-α reached maximal levels on Day 4 in both strains of mice while the peak of IFN-β expression was delayed in BALB/c mice (Fig 5A and 5B). It is of interest that IFN-β was induced in C57BL/6 mice only after coinfection, but not after infection with pneumococci or influenza virus alone. Expression of both type I IFNs was much greater in C57BL/6 mice compared to BALB/c mice. These differences appear to correlate with the differential survival kinetics of the two strains after coinfection (Fig 1). IFN-γ levels increased after Day 4 and continued to increase through Day 8 in both BALB/c (Fig 5C) and C57BL/6 mice (Fig 5F). These data show that type-I IFN was upregulated early during co-infection while type-II IFN was induced later in the infection process.

**Fig 6:**
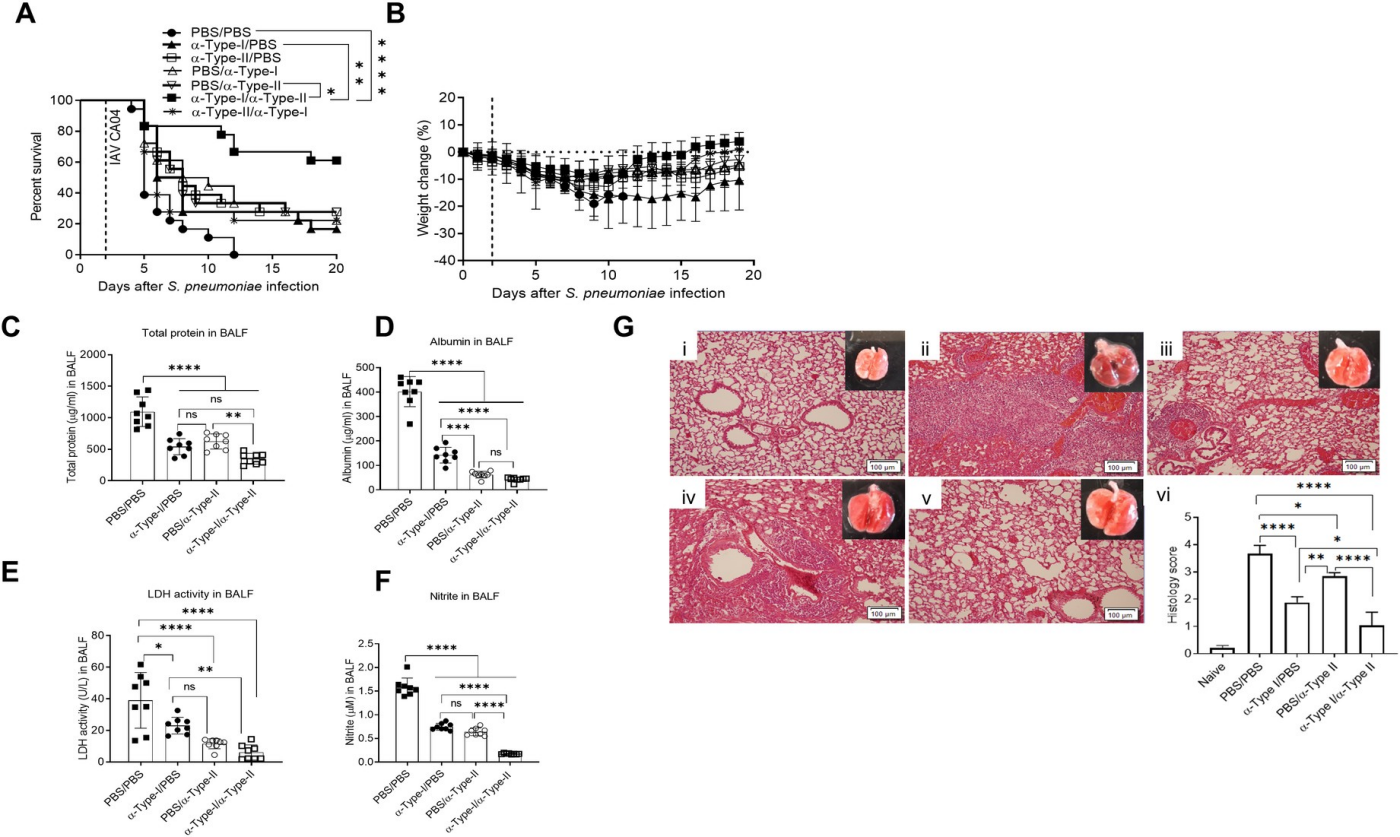
Therapeutic neutralization of IFN-αβ and IFN-γ pathways in pneumococcal-influenza virus superinfected mice. **(A,B)** C57BL/6 mice were infected with pneumococci on Day 0 and influenza virus on Day 2. There were 7 experimental groups (18 mice/group): mice received PBS, α-IFNαβR mAb or α-IFN-γ mAb early after infection (Days 0, 1, 2, and 3) together with PBS, α-IFNαβR mAb or α-IFN-γ mAb late after infection (Days 4, 6, 8, 10 and 12) as indicated in the figure. Survival data were analyzed by log-rank Mantel-Cox test. The data were pooled from two independent experiments. (**C-F**) BALF were analyzed for markers of tissue integrity: (**C**) total protein, (**D**) albumin, (**E**) LDH activity and (**F**) nitrite in the following treatment groups: PBS-PBS, α-Type-I IFN/PBS, PBS/α-Type-II IFN, and α-Type-I IFN/α-Type-II IFN. Data are presented as mean ± SD from 8 mice/group. (**G)** Lung tissue histopathology analysis on Day 5 after co-infection**. (i),** naïve mouse; **(ii**) mouse treated with PBS **(iii)** mouse treated with α-Type-I IFN/PBS; **(iv**) mouse treated with PBS/α-Type-II IFN; and (**v**) mouse treated with α-Type-I IFN/α-Type-II IFN. 20X magnification, scale = 100μm. Also shown is the representative gross pathology of the whole lungs; and **(vi**) Pathology scoring for 4 mice/group. Statistical analyses were performed by two-way ANOVA. ^ns^*P*>0.05; *P<0.05; **P<0.01, ***P<0.001; ****P<0.0001; ns = not significant.

Exploiting the differential kinetics of IFN expression, we next designed a study to determine whether mAb-mediated neutralization of type-I and type-II IFN pathways, either alone or in combination, could be used therapeutically following pneumococcal-influenza superinfection to enhance survival. Anti-IFNαβR mAb alone, anti-IFN-γ mAb alone, or both mAbs were administered to mice over several days, either early after co-infection or at later time points. All PBS-treated control animals died by Day 12 (Fig 6A and 6B). Treatment with one mAb alone either early or late during co-infection resulted in about 20% survival. However, anti-IFNαβR mAb given early after co-infection together with anti-type-II IFN mAb given at later time points increased survival to approximately 60% (Fig 6A and 6B). This therapeutic combination was consistent with the differences in kinetics of cytokine expression seen above. Reversing the sequence (anti-type-II IFN mAb given early and anti-IFNαβR mAb given later) was significantly less effective and resulted in only about 20% survival, similar to what was observed after treatment with either mAb alone.

To investigate the impact of type-I and type-II IFN neutralization on tissue integrity, we assessed total protein, albumin, lactate dehydrogenase (LDH) and nitrite in BALF (Fig 6C–6F). Four animal groups were examined: mice treated with PBS, mice treated with only anti-IFNαβR mAb early, mice treated with only anti-type-II IFN mAb late, or mice treated with both anti-IFNαβR mAb early/anti-type-II IFN mAb late. Compared to PBS-treated mice, all mAb-treated mice showed improved epithelial barrier function as evidenced by lower levels of total protein, albumin, LDH and nitrite in BALF. Consistent with the survival results, mice treated therapeutically with both mAbs showed the least amount of barrier damage.

Gross pathology showed that lung damage was obvious in PBS-treated, superinfected mice (Fig 6G, panel ii), compared to uninfected mice (Fig 6G, panel i). In agreement with the data in Fig 6C–6F, histology scores analyzed on Day 5 after co-infection were lower in mice treated with anti-IFNαβR mAb early or anti-type-II IFN mAb late (Fig 6G, panels iii, iv, and vi). However, the histology scores were lowest in mice treated with a combination of both anti-IFNαβR mAb early/anti-type-II IFN mAb late (Fig 6G, panels v and vi). Mice treated with only PBS began dying shortly thereafter, but we could examine lung inflammation in mAb-treated animals as late as Day 13. Similar to the observations on Day 5, animals treated with a combination of anti-type-I and anti-type-II IFN pathway mAbs exhibited the least amount of tissue damage at this time point (S8 Fig, panels i to vi). Taken together, these results demonstrate the complementary roles of type-I and type-II IFNs in mediating lung tissue damage during bacterial-viral superinfection and the ability of appropriately-timed, combination mAb therapy to prevent such tissue damage.

### Superinfection with colonizing serotype 14 *S*. *pneumoniae* and PR8 influenza virus

**Fig 7:**
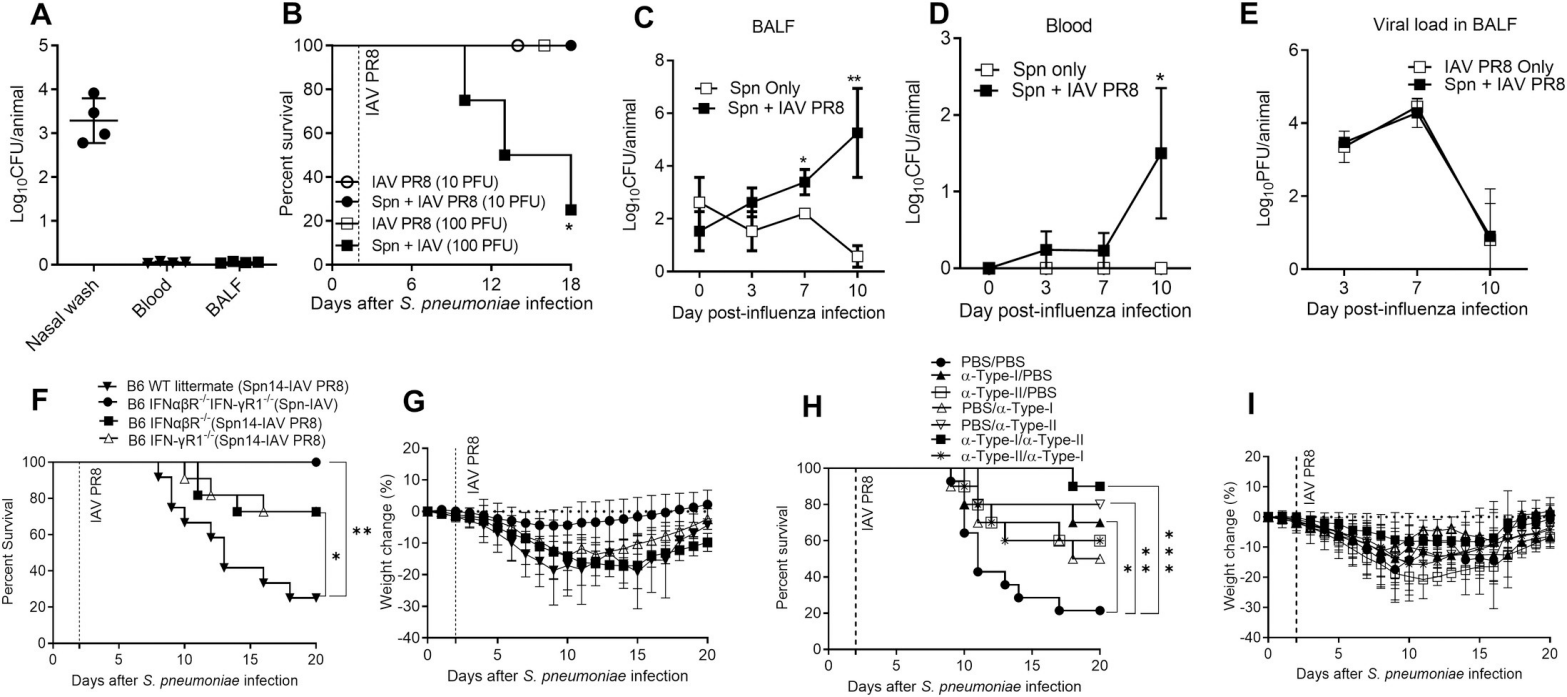
Effects of type-I and type-II IFNs during colonization with serotype-14 *S*. *pneumoniae* followed by superinfection with PR8 influenza virus. **(A)** C57BL/6 mice were colonized with 2x10^6^ CFU of serotype 14 *S*. *pneumoniae* and bacterial burdens in the nasal washes, blood and BALF were determined two days later. (**B**) On Day 2 post-colonization, mice were infected with 10 or 100 PFU of PR8 influenza virus and survival was monitored. Mice were also infected with influenza virus only to ensure that mortality was not due to viral infection. On the indicated days post-influenza virus co-infection, bacterial burdens were assessed in the BALF (**C**) and blood (**D**) and influenza virus titers were determined in the BALF (**E**). A minimum of 4 and 5 mice were used in each experimental group to assess bacterial burden and survival, respectively. Statistical analyses were performed by two-way ANOVA. *P<0.05; **P<0.01. (**F,G**) C57BL/6 WT (n = 12), C57BL/6 IFNαβR^-/^ (n = 11), IFN-γR1^-/-^ (n = 11) and IFNαβR^-/-^IFN-γR1^-/-^ (n = 8) mice were monitored for (**F)** survival and (**G**) weight loss following intranasal infection with 2 x 10^6^ Spn on Day 0 and 100 PFU of PR8 IAV on Day 2. **(H,I)** C57BL/6 WT mice were infected with *S*. *pneumoniae* serotype 14 on Day 0 and influenza virus on Day 2. Mice received PBS, α-IFNαβR mAb or αIFN-γ mAb early after infection (Days 0, 1, 2, and 3) together with PBS, α-IFNαβR mAb or α-IFN-γ mAb late after infection (Days 4, 6, 8, 10 and 12) as indicated in the figure. 10 mice/group, except the PBS control group which included 14 mice. All survival data were analyzed by log-rank Mantel-Cox test. *P<0.05; **P<0.01, ***P<0.001.

It was important to determine whether the above results could be generalized to a different pneumococcal-influenza co-infection model. To test this, we colonized mice with 2x10^6^ CFU of serotype 14 *S*. *pneumoniae* (strain TJ0983) and 48 h later, challenged with 10 or 100 PFU of PR8 virus. Following colonization in the absence of viral challenge, pneumococci were detected only in the nasal wash, and the bacteria had not disseminated to the lung or blood (Fig 7A). After inoculation of 10 PFU PR8 influenza virus, all of the mice survived while 75% of the colonized mice inoculated with 100 PFU of PR8 virus died (Fig 7B), indicating that susceptibility is dependent on the infectious dose. To determine if impaired bacterial or viral clearance caused loss of resistance, BALF and blood were harvested at various time points from mice colonized with *S*. *pneumoniae* only or infected with both pathogens. *S*. *pneumoniae* CFUs were at the lower limit of detection in both the BALF and blood of mice colonized only with pneumococci; however, bacterial outgrowth occurred in co-infected mice on days 7 and 10 in the BALF (Fig 7C) and on Day 10 in the blood (Fig 7D). There were no significant differences in the viral titers between influenza virus-infected and co-infected mice (Fig 7E).

We next tested C57BL/6 WT, IFNαβR^-/-^IFN-γR1^-/-^, IFNαβR^-/-^ and IFN-γR1^-/-^ mice for morbidity and mortality following infection with 2 x 10^6^ CFU of serotype 14 *S*. *pneumoniae* on Day 0 and 100 PFU of PR8 virus on Day 2. While 80% of WT mice succumbed to co-infection (Fig 7F), mice deficient in type-I or type-II IFN signaling pathways demonstrated approximately 28% mortality (Fig 7F and 7G). Mice lacking both IFN pathways demonstrated 100% survival after superinfection (Fig 7F), similar to what was seen above in the serotype 3 pneumococcus-CA04 virus co-infection model. All WT and KO mice infected with either pathogen alone survived. These data confirmed the critical and complementary roles of type-I and type-II IFNs in facilitating susceptibility in bacterial-viral co-infection.

We further explored whether mAb-mediated neutralization of type-I and type-II IFN pathways, either alone or in combination, could be used therapeutically for protection of WT mice against serotype 14 *S*. *pneumoniae*-PR8 virus co-infection. Treatment with anti-IFNαβR mAb alone, anti-IFN-γ mAb alone, or both mAbs was tested either early after co-infection or at later time points, as in our earlier experiments with serotype 3 pneumococcus-CA04 virus superinfection (Fig 6). Approximately 80% of the PBS-treated control mice died by Day 17 (Fig 7H). Treatment with either mAb alone resulted in about 50% survival in all cases. However, anti-IFNαβR mAb given early after co-infection together with anti-IFN-γ mAb given at later time points increased survival to approximately 90% (Fig 7H and 7I). Reversing the sequence (anti-type-II IFN mAb given early and anti-type I mAb given later) was less effective and resulted in only about 60% survival, similar to what was observed following treatment with either mAb alone. Although the differences between groups treated with single mAbs versus both mAbs were not statistically significant, there was clear trend towards delayed mortality and overall increased survival in the group inoculated with IFNαβR mAb early after co-infection together with anti-IFN-γ mAb given later.

## Discussion

Our results demonstate that *in vivo* mAb-mediated neutralization of both type-I and type-II IFN pathways is highly effective in increasing resistance and survival of mice to bacterial-viral superinfection. We used two mouse models of *S*. *pneumoniae* infection in which the bacteria remained in the upper respiratory tract until sublethal influenza virus challenge two days later, which then caused aspiration of the bacteria into the lungs and lethal disease. Neutralization of the type-I IFN pathway early during the co-infection process to prevent virus-induced inflammation was highly effective in decreasing lung damage and enhancing survival, but only when combined with neutralization of type-II IFN later during co-infection to allow effective lung bacterial clearance.

We attempted to mimic human bacterial-viral co-infection using a nasopharyngeal carriage model in which a sublethal dose of *S*. *pneumoniae* was inoculated locally in a low volume to allow colonization of the upper respiratory tract but not infection of the lungs. The animals were then challenged with a sublethal dose of influenza virus two days later, before the bacteria were cleared by the host immune system [21]. Using two different strains of pneumococcus and two different strains of influenza virus, we found that bacteria entered the lung within 48 h after virus co-infection and then caused lung damage, bacteremia and mortality. In healthy humans, *S*. *pneumoniae* often colonize the nasopharynx without causing overt disease. The duration of such colonization in the pediatric population may be as short as 7 days to as long as 50 days, and in adults, may be even shorter [22,23]. Under homeostatic conditions, immunological mechanisms control pneumococcal growth during the colonization/carriage phase but upon influenza infection, this control is lost [24,25]. Similar to the results of our experiments, bacteria spread from the nasopharynx to the surrounding tissue following influenza, ultimately causing bacteremia and severe life-threatening disease [17,26–29].

Synergy between *S*. *pneumoniae* colonization and subsequent viral infection has not been widely studied in animal models. Most mouse models previously used to investigate lethal co-infection, including in our own laboratory, have examined bacterial infection that is initiated following influenza [7,9–12,14,15]. Nevertheless, we found that reversing the order of co-infection to better mimic human superinfection, *i*.*e*., pneumococcal colonization of the upper respiratory tract followed by viral infection, also led to synergistic morbidity and mortality. Although it is generally believed that humans become co-infected through influenza-induced aspiration of colonizing bacteria [30], for convenience, bacteria are typically inoculated in animals directly into the lungs following influenza [9,15]. Use of animal models to understand the pathways responsible for co-infection during influenza has been further complicated by other factors, including: 1) the window of susceptibility to bacterial co-infection in humans is typically seen approximately 7–10 days after influenza virus infection, at a time when the virus is being cleared by the immune system. However, some investigators have studied co-infection of mice at various other times, including bacterial challenges as early as 3 days after viral infection [10,31,32], at a time when viral titers and lung inflammation are peaking, or several months after influenza [33], and 2) mice have been challenged with different quantities of bacteria including very large amounts that are likely not seen in natural cases of human infection, and which overwhelm the protective alveolar macrophage barrier, leading to active recruitment of highly inflammatory neutrophils in a short period of time [31,34,35]. Unlike these studies and most preclinical studies that examine protection against a single infectious disease, sublethal doses of both pathogens were used for co-infections in our current study, again to more realistically mimic human exposure. Mice infected with *S*. *pneumoniae* or influenza virus alone did not develop lethal disease. Instead, superinfection allowed the microbes that by themselves were not virulent, to become highly pathogenic and cause lethal infection in the period of time typically seen in humans. Using this pneumococcal-influenza virus superinfection model, similar results were obtained in both C57BL/6 and BALB/c mice; the experiments were therefore internally controlled for reproducibility.

It is likely that impaired barrier functions and tissue damage following outgrowth of pneumococcus during co-infection is the main cause of lethality. Superinfected mice displayed significantly greater numbers of monocytes, neutrophils and interstitial macrophages, as well as increased levels of inflammatory cytokines, in the pulmonary tract compared to mice infected with either pathogen alone. Increases in inflammatory cell numbers likely contributed to the large amount of tissue damage observed in the superinfected animals. We made the observation that significantly greater numbers of interstitial macrophages were present in the lungs of co-infected mice but not in *S*. *pneumoniae* or H1N1 CA04 singly-infected mice, in agreement with Sabatel and colleagues [36]. Interstitial macrophages are known to secrete IL-1, IL-6, and TNF-α [37–39] and thus, could play a pathogenic role during *S*. *pneumoniae*-influenza superinfection. A previous study reported that monocytes expressing TNF-related apoptosis-inducing ligand cause lung damage and subsequent bacterial superinfection in influenza-*S*. *pneumoniae* co-infection [40]. In that study, bacterial superinfection also led to neutrophil recruitment and TNF-α production, which is in agreement with our results, although the previous study reported that the recruited neutrophils and TNF-α production were beneficial in controlling infection while in our experiments, increased levels of neutrophils and TNF-α correlated with mortality, as was also shown in another report [41]. GM-CSF has been described to have a beneficial role during influenza-*S*. *aureus* co-infection [41]. However, high levels of GM-CSF in co-infected mice in the present study correlated with lethality. In BALB/c mice, levels of alveolar macrophages can decrease during influenza-pneumococcal co-infection [13,42] but we saw no changes in the expression of these cells following pneumococcal-influenza co-infection compared to viral infection alone.

We have now shown for the first time that type-I and type-II IFN pathways act collectively and in unison to mediate increased susceptibility to pneumococcal-influenza superinfection. This was demonstrated using two complementary approaches–KO mice genetically deficient in expression of the two IFN pathways as well as WT mice treated with neutralizing anti-IFN mAbs. It was found that mice lacking both type-I and type-II IFN pathways were significantly better protected from co-infection than mice lacking either pathway alone. Double-deficient animals also showed decreased lung tissue damage as well as lower bacterial burdens in the lungs and bloodstream. Of particular interest, we found that the most improved survival in WT mice was obtained when anti-IFNαβR mAb was injected early during co-infection and anti-type-II IFN mAb was inoculated during later stages of co-infection. This finding agrees with the dynamics of IFN expression seen in our superinfected mice and also with the reported modes of action for each respective cytokine during secondary bacterial infection following influenza. For type-I IFN, the Weiser group [8] has shown that virus-induced type-I IFN in the upper respiratory tract induces inflammation which then allows bacteria in the nasopharynx to enter the lungs. We have now confirmed the ability of type-I IFN-dependent inflammation in the upper respiratory tract to increase bacterial colonization and spread into the lungs. While some groups have reported an important role for type-I IFN in co-infection susceptibility [7–11], others have failed to observe this [15,43]. We believe that these differences could be due to the differential virulence properties of the pathogen strains used as well as the use of deep anesthesia, which could allow the bacteria to be delivered directly into the lungs without a requirement for type-I IFN to accomplish this. Type-II IFN, on the other hand, has been consistently found to directly inhibit macrophage function and maintenance of tissue integrity through constraints on innate lymphoid cytokine expression, inhibition of scavenger receptor expression, and in some mouse strains, increased rates of macrophage cell death [6,8,13,15,18,42]. In addition to the roles of type-I and type-II IFN during superinfection, other groups have described an important contribution for type-III IFN in mediating enhanced susceptibility to co-infections. Although in-depth investigation into all of the contributions of type-I, type-II, and type-III IFN pathways during pneumococcal-influenza superinfection is of interest, such studies would be complex and outside the scope of the current study. Nevertheless, we are currently exploring the possible importance of type III IFN in our pneumococcus-influenza virus co-infection mouse model.

In summary, our findings using murine bacterial colonization followed by influenza resolve a current conundrum in the field regarding the relative importance of type-I and type-II IFNs in bacterial-viral superinfection and have provided new insights into possible host-directed approaches for treatment. Our study provides a novel basis for monitoring of patients for levels of type-I and type-II IFNs during early and late stages of co-infection and for the use of sequential mAb therapeutic strategies for management of bacterial superinfections during influenza.

## Materials and methods

### Ethics statement

Experimental animal protocols were in accordance with the Guide for the Care and Use of the laboratory Animals, NIH. All animal studies were approved by the IACUC of Albany Medical College (Protocol Number: 17–03006 and 20–04001).

### Mice

BALB/c and C57BL/6 WT mice were purchased from Jackson Laboratories (Bar Harbor, ME), as were C57BL/6 IFN-γ^-/-^ (strain 002287), BALB/c IFN-γ^-/-^(strain 002286), C57BL/6 IFN-γR1^-/-^ (strain 003288), C57BL/6 IFNAR^-/-^ (strain 010830), and C57BL/6 IFNAR^-/-^IFN-γR1^-/-^ KO mice (strain 029098**)**. BALB/c IFNAR^-/-^ mice were originally provided by Daniel Portnoy (University of California, Berkeley, CA). All mice were bred at Albany Medical College under specific pathogen-free conditions within individually ventilated cages and adult animals of both sexes were used in the experiments.

### Mouse bacterial-viral co-infection model

Stocks of strain A66.1 *S*. *pneumoniae* serotype 3 and H1N1 A/California/04/2009 (CA04) virus were stored at -80°C until use. Mice were lightly anesthetized with isoflourane and then inoculated intranasally with 10^3^ colony forming units (CFU) of pneumococci in 20 μL of PBS. After 48 h, the mice were again anesthetized with isoflourane and infected intranasally with 50 plaque forming units (PFU) of H1N1 CA04 virus in 40 μL of PBS. Mice infected with pneumococci alone received PBS intranasally on Day 2 rather than virus; mice infected with CA04 alone received PBS intranasally on Day 0. The animals were observed daily for weight loss, clinical signs of disease, and survival for a period of 20 days.

Some experiments tested the possibility that intranasal inoculation of PBS on Day 2 caused washing of bacteria from the upper respiratory tract into the lung independently from viral infection. For this purpose, mice were infected with 20 μL of 10^3^ CFU pneumococci and then 48 h later, were inoculated intranasally with PBS or CA04 virus in a total volume of 10, 20, or 40 μL. The animals were sacrificed either within 5 min or 48 h later, and analyzed for bacterial burden in the respiratory tract.

In further experiments, serotype 14 *S*. *pneumoniae* (strain TJ0983) and PR8 (A/Puerto Rico/8/34-H1N1) influenza virus were used to establish a second co-infection model. As above, lightly anesthetized mice were first inoculated intranasally with 20 μL of PBS containing 2x10^6^ CFU of serotype 14 *S*. *pneumoniae*. Two days later, anesthetized mice were infected with either 10 or 100 PFU of PR8 influenza virus in 40 μL of PBS. Control animals were treated with equivalent volumes of PBS.

### Bacterial and viral burden analyses

To measure bacterial burden, mice were bled from the retro-orbital plexus and then euthanized with sodium pentobarbitol to collect nasal washes and lungs. A cannulated needle was inserted into the trachea and nasal washes were collected in 1 ml PBS. Lungs were mechanically homogenized in 1 ml of PBS. The collected samples were then serially diluted and plated on blood agar plates for enumeration of CFU. Viral burdens in lung homogenates were determined by plaque assay on Madin–Darby canine kidney cells.

### Flow cytometry analysis

Single cell suspensions from lungs and bronchoalveolar lavage (BAL) were incubated with FcγRII/III block (2.4G2 mAb) for 15 min and then stained with anti-mouse mAbs followed by staining with Fixable Viability Dye (eFluor 780; eBioscience) to differentiate live and dead cells. Stained cells were analyzed on a BD FACS Canto or BD LSR II using FACSDiva and FlowJo software for data analysis. The mAbs used for staining were anti-CD3 (clone 17A2, APC, BioLegend), anti-CD11b (clone M1/70, PerCP-Cy5.5, BD Biosciences), anti-CD11c (clone N418, Pac Blue, BioLegend), anti-Siglec-F (clone E50-2440, PE, BD Biosciences), anti-MHC class-II (clone M5/114.15.2, Pac Orange, BioLegend), anti-Ly6C (clone HK 1.4, APC, eBioscience), anti-Ly6G (clone 1A8, FITC, BD Biosciences), anti-CD4 (clone GK 1.5, FITC, BioLegend), anti-CD8 (clone 53–6.7, PE, BD Biosciences), and anti-Dx5 (clone DX5, Pac Blue, BioLegend).

### Cytokine analysis

Cytokines in BAL fluid (BALF) were analyzed by a Luminex multiplex bead-based assay (Bio-Rad Laboratories Inc, USA). Type-I IFNs were measured in whole lungs by quantitative reverse transcriptase PCR (RT-qPCR) using Qiagen RNeasy Plus kits. The relative gene abundance was calculated using the ΔΔCt method with β2 microglobulin (β2M) as a housekeeping control. Primers were, IFN-α: 5′-CTGGCCAACCTGCTCTCTAG-3′, 3′-CTCCTGCGGGAATCCAAAGT-5′, IFN-β: 5′-CAGCCTGGCTT CCATCATGA-3′, 3′-TTCCATTCAGCTGCTCCAGG-5′. Levels of IFN-γ in BALF were determined using an IFN-γ ELISA kit per the manufacturer’s protocol (R&D Systems).

### Histopathology analysis

For analysis of lung pathology, lung tissues were fixed in 10% formalin and 5 μm sections were prepared and stained with hematoxylin and eosin (H&E). A scoring system was used based upon previous influenza studies [44–46]. Ten fields of lung sections were randomly chosen and scored as previously described [47]. Images were captured using an Olympus BX41 microscope under 20X magnification and CellSense software. Total protein concentrations in BALF were analyzed using a Pierce BCA protein assay kit (Thermo Scientific). Nitrite levels were quantified using a Griess kit (Life Technologies). The BCG albumin assay kit (Sigma-Aldrich) was used to determine albumin and the Lactate Dehydrogenase (LDH) Activity Assay Kit (Sigma-Aldrich) was used to assess LDH levels.

### *In vivo* mAb neutralization

The mouse type-I and type-II IFN pathways were neutralized by intraperitoneal inoculation of 500 μg/mouse of MAR1-5A3 anti-IFNαβR and 600 μg/mouse of XMG1.2 anti-IFNγ mAbs (BioXCell), in 200 μL PBS/day. The mAbs were injected on Days 0, 1, 2, and 3 and/or Days 4, 6, 8, 10 and 12 after pneumococcal infection, as specified in the Results section. All treatments were carried out in a double-blind manner using mAb preparations coded by an independent investigator. Mortality and weight loss were monitored daily until day 20.

### Statistical analysis

All statistical analyses were performed using GraphPad Prism version 8.0 (Graphpad Software). Kaplan-Meier survival curves were analyzed using the log-rank Mantel-Cox test and weight loss data were analyzed using a two-tailed Mann Whitney U test. Protein expression and histology between multiple groups was analyzed by two-way ANOVA with Bonferroni’s multiple comparisons. *P*<0.05 was considered to be statistically significant.

## Supporting information

S1 Fig Influence of different virus inoculum volumes on bacterial spread. Influenza virus or PBS was inoculated intranasally to colonized mice in volumes of 10, 20, or 40 μL and lung bacterial loads were assessed within 5 min (**A**) or 48 h (**B**). Statistical analyses were performed by two-way ANOVA. *P*>0.05; ns = not significant.
(TIF)

S2 Fig Influenza-induced influx of inflammatory neutrophils into the nasal cavity. (**A**) Protocol for infection and nasal wash sampling. (**B**) Levels of Ly6G^+^CD45^+^ neutrophils in naïve mice, Spn-colonized mice, IAV-infected mice, and co-infected mice. Statistical significance was assessed using the two-way ANOVA. ** indicates *P*<0.01. (C) Representative flow cytometry histogram depicting the Ly6G^+^CD45^+^ neutrophil population in the nasal wash of an IAV-infected mice. (D) Representative immunofluorescence image of Ly6G^+^ neutrophils in an IAV-infected mouse. Ten microliter sample of nasal wash was spread onto a glass slide, fixed and stained with DAPI blue (i), anti-Ly6G mAb, green (ii) and both (iii).
(TIF)

S3 Fig Bacterial burdens of nasal wash of *S*. *pneumoniae* alone (Spn-PBS) and co-infected (Spn-IAV) mice. (**A,B**) Statistical analysis of nasal wash bacterial burdens in mice infected with *S*. *pneumoniae* alone (Spn-PBS), co-infected with serotype 3 *S*. *pneumoniae* and CA04 IAV (Spn-IAV) on Days 4, 6, and 8. The results show an increase in bacterial colonization on Days 6 and 8 following IAV co-infection but no changes in bacterial levels within each group over time. Statistical analyses were performed by two-way ANOVA. *P<0.05; ***P<0.001; ****P<0.0001; ns = not significant.
(TIF)

S4 Fig Gating strategies for (A) myeloid cells and (B) lymphoid cells in BALB/c mice after infection.
(TIF)

S5 Fig Susceptibility of BALB/c mice deficient in the type-I IFN pathway to pneumococcal-influenza virus superinfection. **(A, B)**, BALB/c WT and IFNαβR^-/-^ mice were infected on Day 0 with Spn alone, on Day 2 with IAV alone, or co-infected on the indicated days and monitored for (**A)** survival and (**B**) weight loss. 4–7 co-infected mice/group; 2–4 singly-infected mice/group. Survival data were analyzed by log-rank Mantel-Cox test. *P<0.05.
(TIF)

S6 Fig Susceptibility of C57BL/6 and BALB/c mice deficient in type-II IFN to pneumococcal-influenza virus superinfection. **(A, B)**, C57BL/6 WT and IFN-γ^-/-^ mice were infected on Day 0 with Spn alone, on Day 2 with IAV alone, or co-infected on the indicated days and monitored for (**A)** survival and (**B**) weight loss. 6–7 co-infected mice/group; 4 singly-infected mice/group. **(C, D)**, BALB/c WT and IFN-γ^-/-^ mice were infected on Day 0 with Spn alone, on Day 2 with IAV alone, or co-infected on the indicated days and monitored for (**C)** survival and (D) weight loss. 5 co-infected mice/group; 3 singly-infected mice/group. Survival data were analyzed by log-rank Mantel-Cox test. *P<0.05; **P<0.01; ***P<0.001.
(TIF)

S7 Fig Bacterial burdens in nasal washes and lungs of IFNαβR^-/-^, IFN-γR1^-/-^ and IFNαβR^-/-^IFN-γR1^-/-^ mice after infection with *S*. *pneumoniae* alone (Spn-PBS) or *S*. *pneumoniae*-influenza virus co-infection (Spn-IAV). Nasal washes (**A**) and lung tissues (**B**) were analyzed on Day 7 after infection. Statistical analyses were performed by two-way ANOVA. *P<0.05; ***P<0.001; ****P<0.0001; ns = not significant.
(TIF)

S8 Fig Lung tissue histopathology analysis after completion of treatment. **(i)** naïve mous**e; (ii**) mouse treated with PBS and analyzed on Day 5 after co-infection; **(iii)** mouse treated with α-Type-I IFN/PBS and analyzed on Day 13 after co-infection; **(iv**) mouse treated with PBS/α-Type-II IFN and analyzed on Day 13 after co-infection; and (**v**) mouse treated with α-Type-I IFN/α-Type-II IFN and analyzed on Day 13 after co-infection. 20X magnification, scale = 100μm. Also shown is the representative gross pathology of the whole lungs; and **(vi**) Pathology scoring for 4 mice/group. Statistical analyses were performed by two-way ANOVA. **P<0.01, ****P<0.0001; ns = not significant.
(TIF)

## References

[1] Falsey AR, Becker KL, Swinburne AJ, Nylen ES, Formica MA, Hennessey PA, et al. Bacterial complications of respiratory tract viral illness: a comprehensive evaluation. J Infect Dis. 2013;208(3):432–41. Epub 2013/05/11. 10.1093/infdis/jit190 23661797 PMC3699009

[2] Morens DM, Taubenberger JK, Fauci AS. Predominant role of bacterial pneumonia as a cause of death in pandemic influenza: implications for pandemic influenza preparedness. J Infect Dis. 2008;198(7):962–70. Epub 2008/08/20. 10.1086/591708 18710327 PMC2599911

[3] Gill JR, Sheng ZM, Ely SF, Guinee DG, Beasley MB, Suh J, et al. Pulmonary pathologic findings of fatal 2009 pandemic influenza A/H1N1 viral infections. Arch Pathol Lab Med. 2010;134(2):235–43. Epub 2010/02/04. 10.1043/1543-2165-134.2.235 20121613 PMC2819217

[4] Louria DB, Blumenfeld HL, Ellis JT, Kilbourne ED, Rogers DE. Studies on influenza in the pandemic of 1957–1958. II. Pulmonary complications of influenza. J Clin Invest. 1959;38(1 Part 2):213–65. Epub 1959/01/01. 10.1172/JCI103791 13620784 PMC444127

[5] Grousd JA, Rich HE, Alcorn JF. Host-pathogen interactions in gram-positive bacterial pneumonia. Clin Microbiol Rev. 2019;32(3). Epub 2019/05/31. 10.1128/CMR.00107-18 31142498 PMC6589866

[6] Metzger DW, Sun K. Immune dysfunction and bacterial coinfections following influenza. J Immunol. 2013;191(5):2047–52. Epub 2013/08/22. 10.4049/jimmunol.1301152 23964104 PMC3760235

[7] Lee B, Robinson KM, McHugh KJ, Scheller EV, Mandalapu S, Chen C, et al. Influenza-induced type I interferon enhances susceptibility to gram-negative and gram-positive bacterial pneumonia in mice. Am J Physiol Lung Cell Mol Physiol. 2015;309(2):L158–67. Epub 2015/05/24. 10.1152/ajplung.00338.2014 26001778 PMC4504975

[8] Nakamura S, Davis KM, Weiser JN. Synergistic stimulation of type I interferons during influenza virus coinfection promotes *Streptococcus pneumoniae* colonization in mice. J Clin Invest. 2011;121(9):3657–65. Epub 2011/08/16. 10.1172/JCI57762 21841308 PMC3163966

[9] Shahangian A, Chow EK, Tian X, Kang JR, Ghaffari A, Liu SY, et al. Type I IFNs mediate development of postinfluenza bacterial pneumonia in mice. J Clin Invest. 2009;119(7):1910–20. Epub 2009/06/03. 10.1172/JCI35412 19487810 PMC2701856

[10] Shepardson KM, Larson K, Morton RV, Prigge JR, Schmidt EE, Huber VC, et al. Differential type I interferon signaling is a master regulator of susceptibility to postinfluenza bacterial superinfection. mBio. 2016;7(3). Epub 2016/05/05. 10.1128/mBio.00506-16 27143388 PMC4959663

[11] Shirey KA, Perkins DJ, Lai W, Zhang W, Fernando LR, Gusovsky F, et al. Influenza "trains" the host for enhanced susceptibility to secondary bacterial infection. mBio. 2019;10(3). Epub 2019/05/09. 10.1128/mBio.00810-19 31064834 PMC6509193

[12] Breslow-Deckman JM, Mattingly CM, Birket SE, Hoskins SN, Ho TN, Garvy BA, et al. Linezolid decreases susceptibility to secondary bacterial pneumonia postinfluenza infection in mice through its effects on IFN-gamma. J Immunol. 2013;191(4):1792–9. Epub 2013/07/09. 10.4049/jimmunol.1300180 23833238 PMC3751392

[13] Califano D, Furuya Y, Metzger DW. Effects of influenza on alveolar macrophage viability are dependent on mouse genetic strain. J Immunol. 2018;201(1):134–44. Epub 2018/05/16. 10.4049/jimmunol.1701406 29760191 PMC6008236

[14] Hang do TT, Choi EJ, Song JY, Kim SE, Kwak J, Shin YK. Differential effect of prior influenza infection on alveolar macrophage phagocytosis of *Staphylococcus aureus* and Escherichia coli: involvement of interferon-gamma production. Microbiol Immunol. 2011;55(11):751–9. Epub 2011/09/08. 10.1111/j.1348-0421.2011.00383.x . 21895747

[15] Sun K, Metzger DW. Inhibition of pulmonary antibacterial defense by interferon-gamma during recovery from influenza infection. Nat Med. 2008;14(5):558–64. Epub 2008/04/29. 10.1038/nm1765 . 18438414

[16] Sun K, Ye J, Perez DR, Metzger DW. Seasonal FluMist vaccination induces cross-reactive T cell immunity against H1N1 (2009) influenza and secondary bacterial infections. J Immunol. 2011;186(2):987–93. Epub 2010/12/17. 10.4049/jimmunol.1002664 . 21160043

[17] Weiser JN, Ferreira DM, Paton JC. *Streptococcus pneumoniae*: transmission, colonization and invasion. Nat Rev Microbiol. 2018;16(6):355–67. Epub 2018/03/31. 10.1038/s41579-018-0001-8 29599457 PMC5949087

[18] Califano D, Furuya Y, Roberts S, Avram D, McKenzie ANJ, Metzger DW. IFN-gamma increases susceptibility to influenza A infection through suppression of group II innate lymphoid cells. Mucosal Immunol. 2018;11(1):209–19. Epub 2017/05/18. 10.1038/mi.2017.41 28513592 PMC5693789

[19] Graham MB, Dalton DK, Giltinan D, Braciale VL, Stewart TA, Braciale TJ. Response to influenza infection in mice with a targeted disruption in the interferon gamma gene. J Exp Med. 1993;178(5):1725–32. Epub 1993/11/01. 10.1084/jem.178.5.1725 8228818 PMC2191239

[20] Simell B, Auranen K, Kayhty H, Goldblatt D, Dagan R, O’Brien KL, et al. The fundamental link between pneumococcal carriage and disease. Expert Rev Vaccines. 2012;11(7):841–55. Epub 2012/08/24. 10.1586/erv.12.53 . 22913260

[21] Sun K, Johansen FE, Eckmann L, Metzger DW. An important role for polymeric Ig receptor-mediated transport of IgA in protection against *Streptococcus pneumoniae* nasopharyngeal carriage. J Immunol. 2004;173(7):4576–81. Epub 2004/09/24. 10.4049/jimmunol.173.7.4576 . 15383591

[22] Abdullahi O, Karani A, Tigoi CC, Mugo D, Kungu S, Wanjiru E, et al. Rates of acquisition and clearance of pneumococcal serotypes in the nasopharynges of children in Kilifi District, Kenya. J Infect Dis. 2012;206(7):1020–9. Epub 2012/07/26. 10.1093/infdis/jis447 22829650 PMC3433858

[23] Shak JR, Vidal JE, Klugman KP. Influence of bacterial interactions on pneumococcal colonization of the nasopharynx. Trends Microbiol. 2013;21(3):129–35. Epub 2013/01/01. 10.1016/j.tim.2012.11.005 23273566 PMC3729046

[24] Jochems SP, Marcon F, Carniel BF, Holloway M, Mitsi E, Smith E, et al. Inflammation induced by influenza virus impairs human innate immune control of pneumococcus. Nat Immunol. 2018;19(12):1299–308. Epub 2018/10/31. 10.1038/s41590-018-0231-y 30374129 PMC6241853

[25] Wang XY, Kilgore PE, Lim KA, Wang SM, Lee J, Deng W, et al. Influenza and bacterial pathogen coinfections in the 20th century. Interdiscip Perspect Infect Dis. 2011;2011:146376. Epub 2011/07/13. 10.1155/2011/146376 21747847 PMC3124839

[26] Austrian R. Some aspects of the pneumococcal carrier state. J Antimicrob Chemother. 1986;18 Suppl A:35–45. Epub 1986/07/01. 10.1093/jac/18.supplement_a.35 . 3745031

[27] Bogaert D, De Groot R, Hermans PW. *Streptococcus pneumoniae* colonisation: the key to pneumococcal disease. Lancet Infect Dis. 2004;4(3):144–54. Epub 2004/03/05. 10.1016/S1473-3099(04)00938-7 . 14998500

[28] Jia L, Xie J, Zhao J, Cao D, Liang Y, Hou X, et al. Mechanisms of severe mortality-associated bacterial co-infections following influenza virus infection. Front Cell Infect Microbiol. 2017;7:338. Epub 2017/08/22. 10.3389/fcimb.2017.00338 28824877 PMC5540941

[29] Musher DM. How contagious are common respiratory tract infections? N Engl J Med. 2003;348(13):1256–66. Epub 2003/03/28. 10.1056/NEJMra021771 . 12660390

[30] Lijek RS, Weiser JN. Co-infection subverts mucosal immunity in the upper respiratory tract. Curr Opin Immunol. 2012;24(4):417–23. Epub 2012/06/05. 10.1016/j.coi.2012.05.005 22658762 PMC3423578

[31] McNamee LA, Harmsen AG. Both influenza-induced neutrophil dysfunction and neutrophil-independent mechanisms contribute to increased susceptibility to a secondary *Streptococcus pneumoniae* infection. Infect Immun. 2006;74(12):6707–21. Epub 2006/09/20. 10.1128/IAI.00789-06 16982840 PMC1698099

[32] Wang X, Yuan J, Wang H, Gan N, Zhang Q, Liu B, et al. Progranulin decreases susceptibility to *Streptococcus pneumoniae* in influenza and protects against lethal coinfection. J Immunol. 2019;203(8):2171–82. Epub 2019/09/15. 10.4049/jimmunol.1900248 . 31519865

[33] Didierlaurent A, Goulding J, Patel S, Snelgrove R, Low L, Bebien M, et al. Sustained desensitization to bacterial Toll-like receptor ligands after resolution of respiratory influenza infection. J Exp Med. 2008;205(2):323–9. Epub 2008/01/30. 10.1084/jem.20070891 18227219 PMC2271005

[34] Kohler J, Breitbach K, Renner C, Heitsch AK, Bast A, van Rooijen N, et al. NADPH-oxidase but not inducible nitric oxide synthase contributes to resistance in a murine *Staphylococcus aureus* Newman pneumonia model. Microbes Infect. 2011;13(11):914–22. Epub 2011/06/04. 10.1016/j.micinf.2011.05.004 . 21635963

[35] van der Sluijs KF, Nijhuis M, Levels JH, Florquin S, Mellor AL, Jansen HM, et al. Influenza-induced expression of indoleamine 2,3-dioxygenase enhances interleukin-10 production and bacterial outgrowth during secondary pneumococcal pneumonia. J Infect Dis. 2006;193(2):214–22. Epub 2005/12/20. 10.1086/498911 . 16362885

[36] Sabatel C, Radermecker C, Fievez L, Paulissen G, Chakarov S, Fernandes C, et al. Exposure to bacterial cpg DNA protects from airway allergic inflammation by expanding regulatory lung interstitial macrophages. Immunity. 2017;46(3):457–73. Epub 2017/03/23. 10.1016/j.immuni.2017.02.016 . 28329706

[37] Franke-Ullmann G, Pfortner C, Walter P, Steinmuller C, Lohmann-Matthes ML, Kobzik L. Characterization of murine lung interstitial macrophages in comparison with alveolar macrophages in vitro. J Immunol. 1996;157(7):3097–104. Epub 1996/10/01. . 8816420

[38] Kawano H, Kayama H, Nakama T, Hashimoto T, Umemoto E, Takeda K. IL-10-producing lung interstitial macrophages prevent neutrophilic asthma. Int Immunol. 2016;28(10):489–501. Epub 2016/03/16. 10.1093/intimm/dxw012 . 26976823

[39] Wizemann TM, Laskin DL. Enhanced phagocytosis, chemotaxis, and production of reactive oxygen intermediates by interstitial lung macrophages following acute endotoxemia. Am J Respir Cell Mol Biol. 1994;11(3):358–65. Epub 1994/09/01. 10.1165/ajrcmb.11.3.8086172 . 8086172

[40] Ellis GT, Davidson S, Crotta S, Branzk N, Papayannopoulos V, Wack A. TRAIL+ monocytes and monocyte-related cells cause lung damage and thereby increase susceptibility to influenza-*Streptococcus pneumoniae* coinfection. EMBO Rep. 2015;16(9):1203–18. Epub 2015/08/13. 10.15252/embr.201540473 26265006 PMC4576987

[41] Subramaniam R, Barnes PF, Fletcher K, Boggaram V, Hillberry Z, Neuenschwander P, et al. Protecting against post-influenza bacterial pneumonia by increasing phagocyte recruitment and ROS production. J Infect Dis. 2014;209(11):1827–36. Epub 2013/12/25. 10.1093/infdis/jit830 . 24367039

[42] Ghoneim HE, Thomas PG, McCullers JA. Depletion of alveolar macrophages during influenza infection facilitates bacterial superinfections. J Immunol. 2013;191(3):1250–9. Epub 2013/06/28. 10.4049/jimmunol.1300014 23804714 PMC4907362

[43] Stegemann-Koniszewski S, Gereke M, Orrskog S, Lienenklaus S, Pasche B, Bader SR, et al. TLR7 contributes to the rapid progression but not to the overall fatal outcome of secondary pneumococcal disease following influenza A virus infection. J Innate Immun. 2013;5(1):84–96. Epub 2012/11/17. 10.1159/000345112 23154432 PMC6741512

[44] Fukushi M, Ito T, Oka T, Kitazawa T, Miyoshi-Akiyama T, Kirikae T, et al. Serial histopathological examination of the lungs of mice infected with influenza A virus PR8 strain. PLoS One. 2011;6(6):e21207. Epub 2011/06/28. 10.1371/journal.pone.0021207 21701593 PMC3118813

[45] Gibson-Corley KN, Olivier AK, Meyerholz DK. Principles for valid histopathologic scoring in research. Vet Pathol. 2013;50(6):1007–15. Epub 2013/04/06. 10.1177/0300985813485099 23558974 PMC3795863

[46] Taubenberger JK, Morens DM. The pathology of influenza virus infections. Annu Rev Pathol. 2008;3:499–522. Epub 2007/11/28. 10.1146/annurev.pathmechdis.3.121806.154316 18039138 PMC2504709

[47] Buchweitz JP, Karmaus PW, Harkema JR, Williams KJ, Kaminski NE. Modulation of airway responses to influenza A/PR/8/34 by Delta9-tetrahydrocannabinol in C57BL/6 mice. J Pharmacol Exp Ther. 2007;323(2):675–83. Epub 2007/08/30. 10.1124/jpet.107.124719 . 17726158

