# Supplementary figures and images for: Sequential targeting of interferon pathways for increased host resistance to bacterial superinfection during influenza
Source: PLoS Pathog. 2021 Mar 9;17(3):e1009405. doi: 10.1371/journal.ppat.1009405 (PMC7978370; doi:10.1371/journal.ppat.1009405)

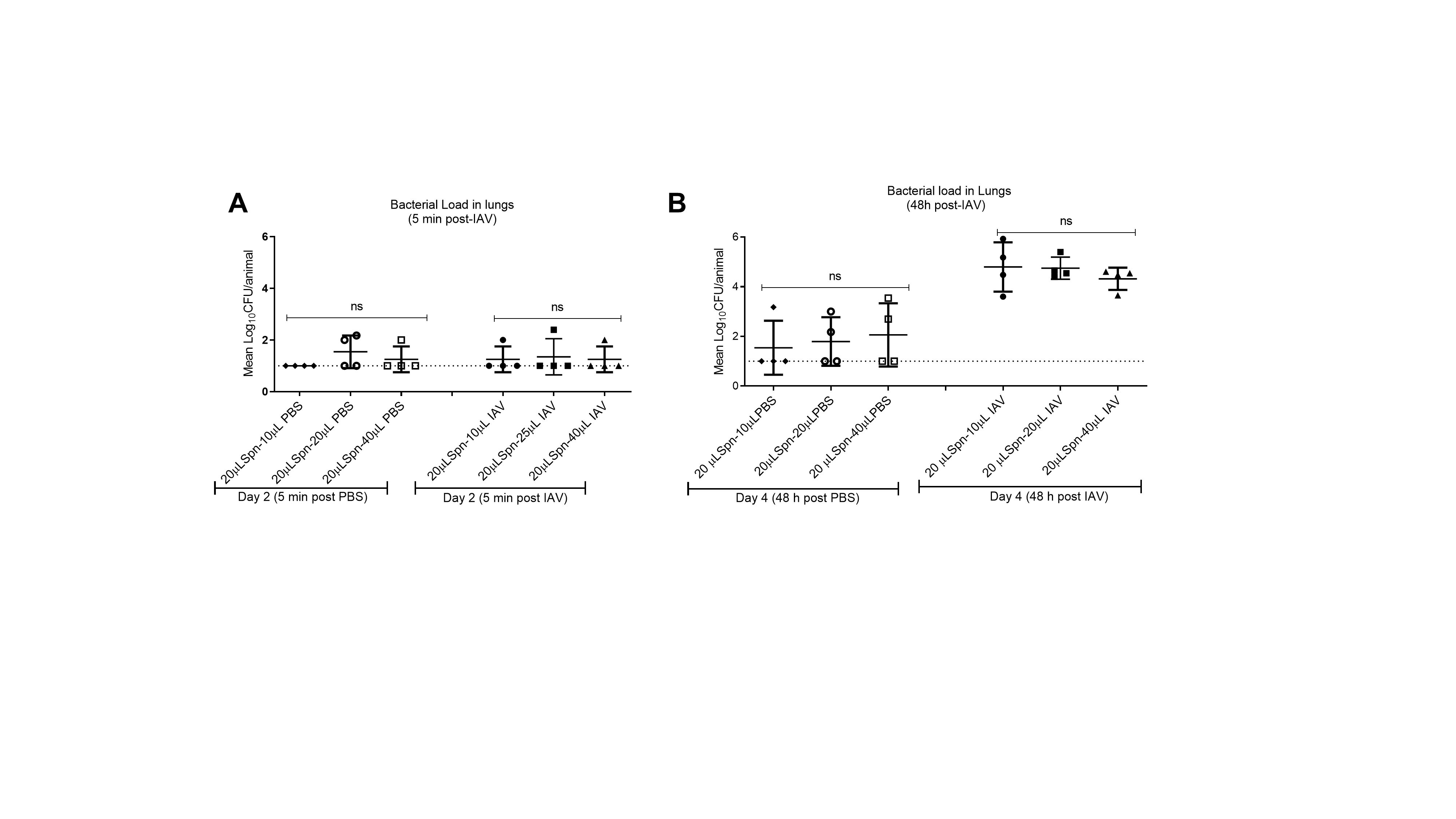

Supplement: S1 Fig — Influenza virus or PBS was inoculated intranasally to colonized mice in volumes of 10, 20, or 40 μL and lung bacterial loads were assessed within 5 min (A) or 48 h (B). Statistical analyses were performed by two-way ANOVA. P>0.05; ns = not significant. (TIF) [file ppat.1009405.s001.TIF]

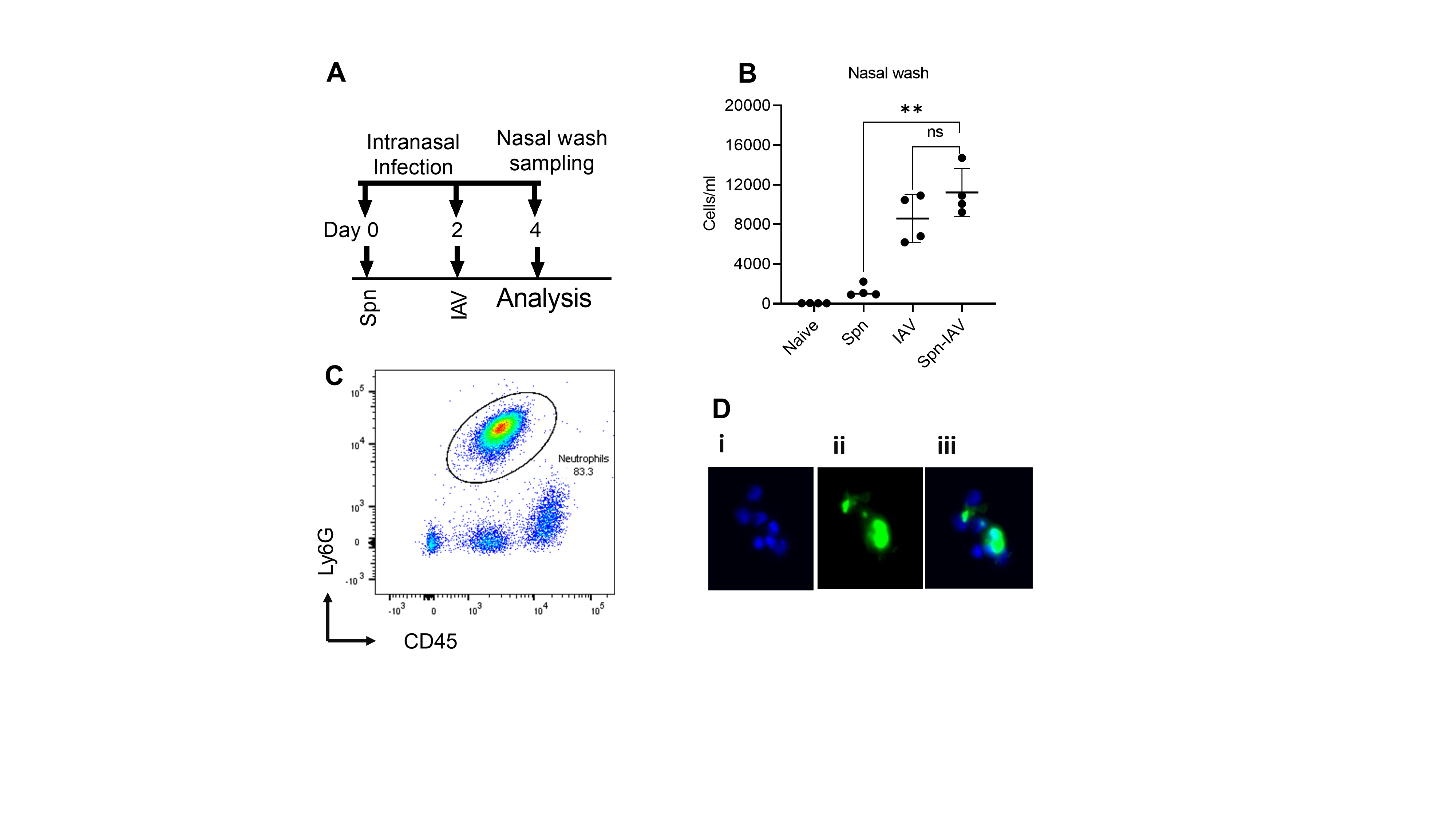

Supplement: S2 Fig — (A) Protocol for infection and nasal wash sampling. (B) Levels of Ly6G+CD45+ neutrophils in naïve mice, Spn-colonized mice, IAV-infected mice, and co-infected mice. Statistical significance was assessed using the two-way ANOVA. ** indicates P<0.01. (C) Representative flow cytometry histogram depicting the Ly6G+CD45+ neutrophil population in the nasal wash of an IAV-infected mice. (D) Representative immunofluorescence image of Ly6G+ neutrophils in an IAV-infected mouse. Ten microliter sample of nasal wash was spread onto a glass slide, fixed and stained with DAPI blue (i), anti-Ly6G mAb, green (ii) and both (iii). (TIF) [file ppat.1009405.s002.TIF]

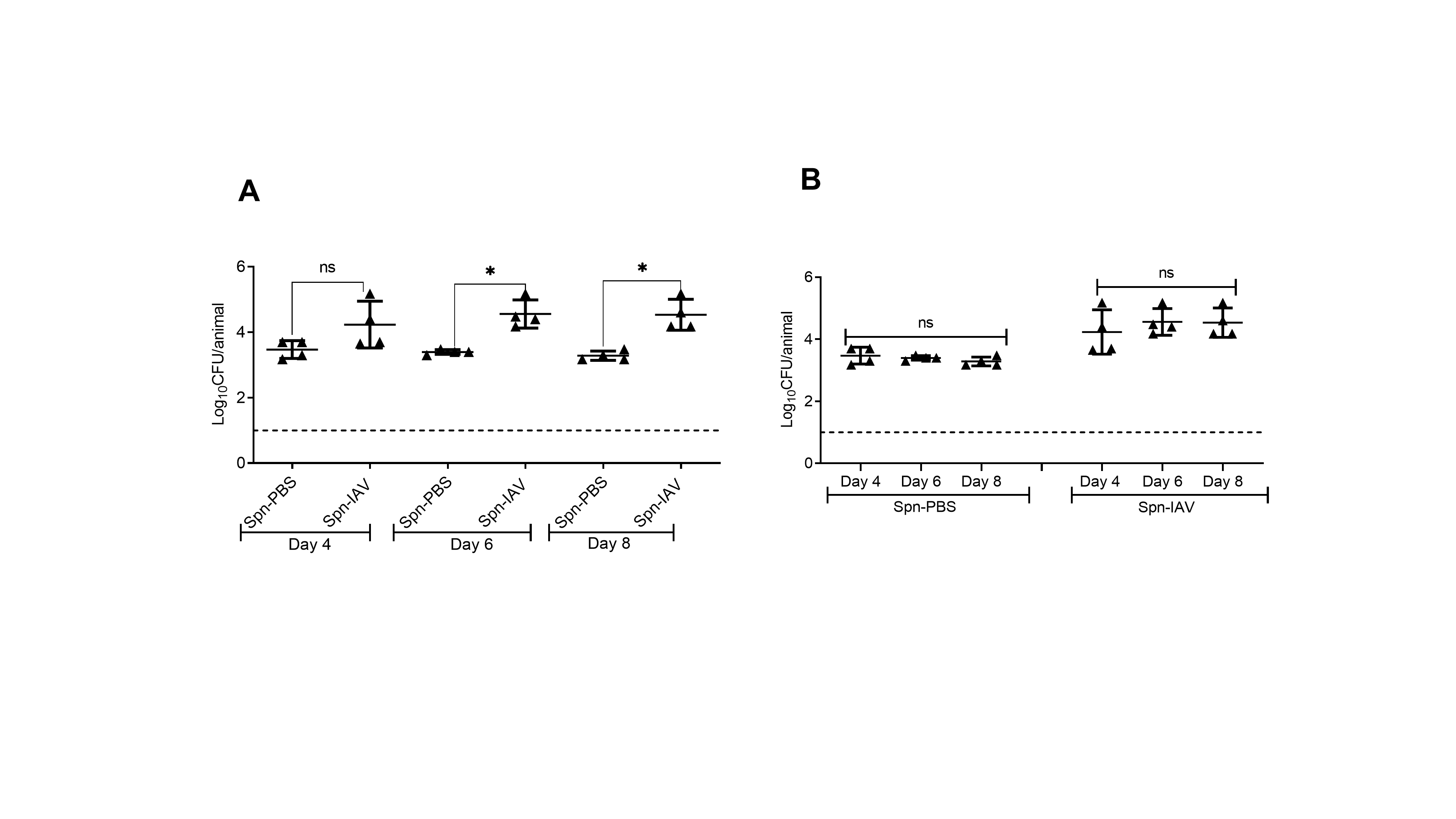

Supplement: S3 Fig — (A,B) Statistical analysis of nasal wash bacterial burdens in mice infected with S. pneumoniae alone (Spn-PBS), co-infected with serotype 3 S. pneumoniae and CA04 IAV (Spn-IAV) on Days 4, 6, and 8. The results show an increase in bacterial colonization on Days 6 and 8 following IAV co-infection but no changes in bacterial levels within each group over time. Statistical analyses were performed by two-way ANOVA. *P<0.05; ***P<0.001; ****P<0.0001; ns = not significant. (TIF) [file ppat.1009405.s003.TIF]

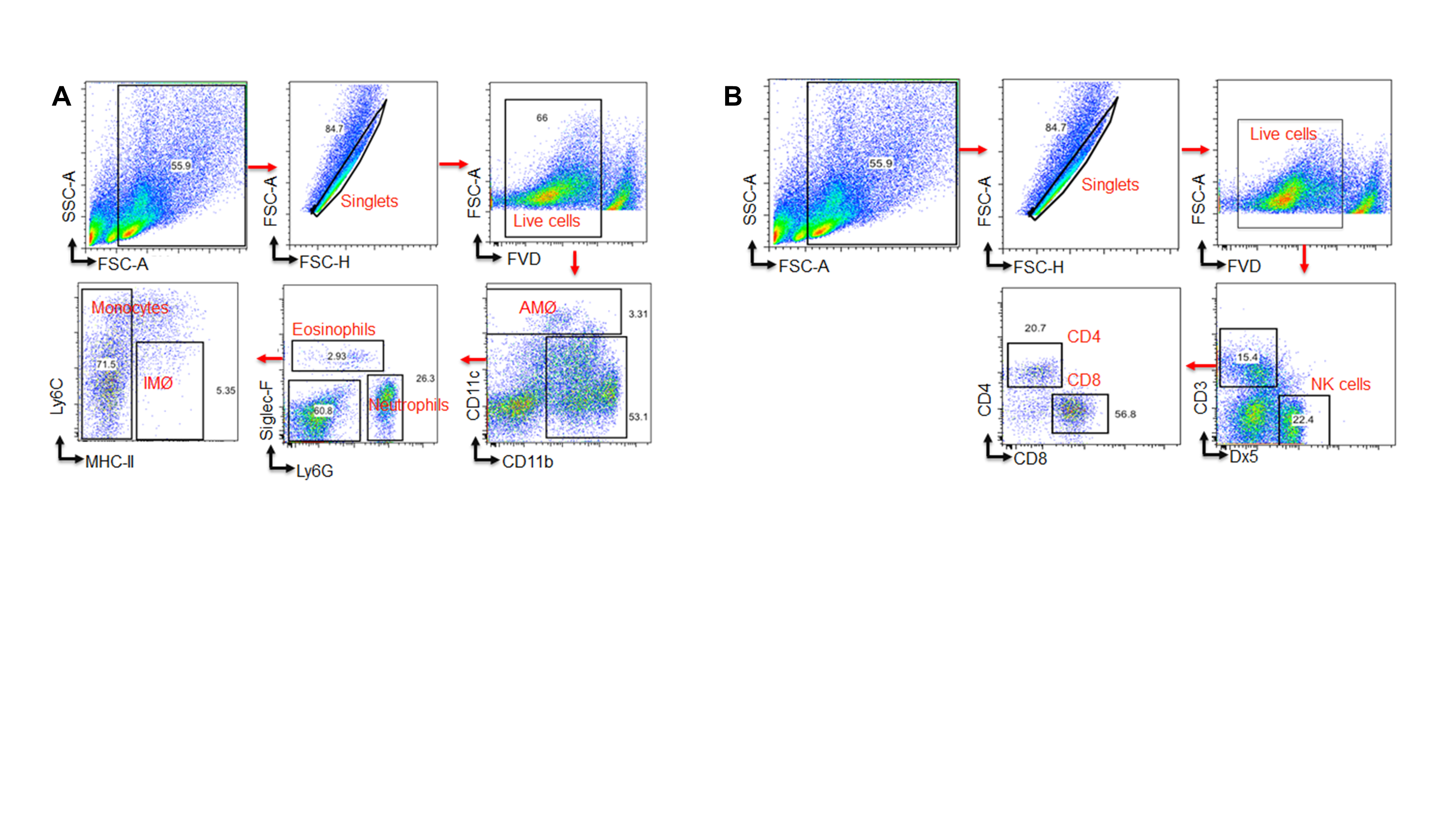

Supplement: S4 Fig — Gating strategies for (A) myeloid cells and (B) lymphoid cells in BALB/c mice after infection. (TIF) [file ppat.1009405.s004.TIF]

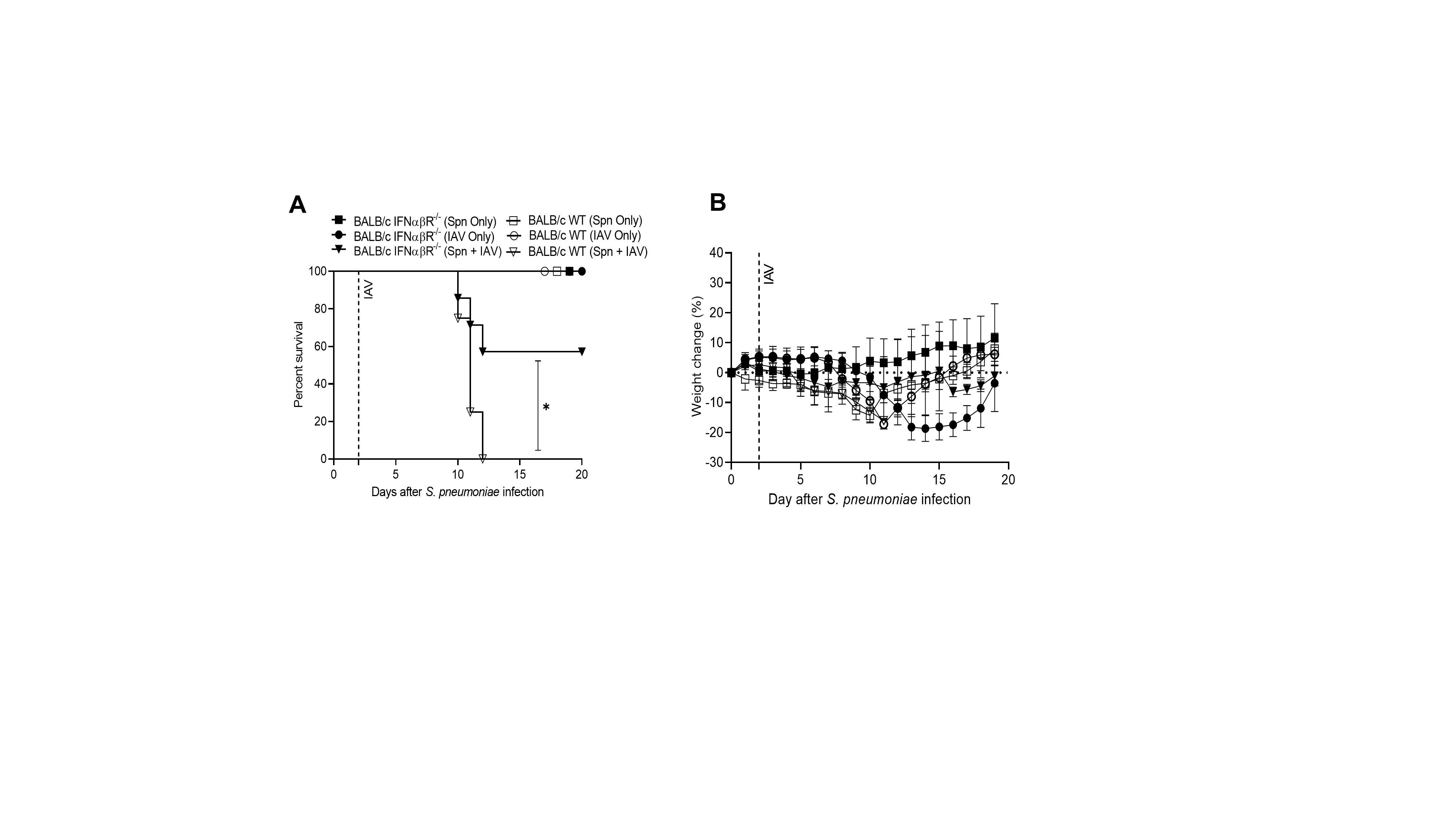

Supplement: S5 Fig — (A, B), BALB/c WT and IFNαβR-/- mice were infected on Day 0 with Spn alone, on Day 2 with IAV alone, or co-infected on the indicated days and monitored for (A) survival and (B) weight loss. 4–7 co-infected mice/group; 2–4 singly-infected mice/group. Survival data were analyzed by log-rank Mantel-Cox test. *P<0.05. (TIF) [file ppat.1009405.s005.TIF]

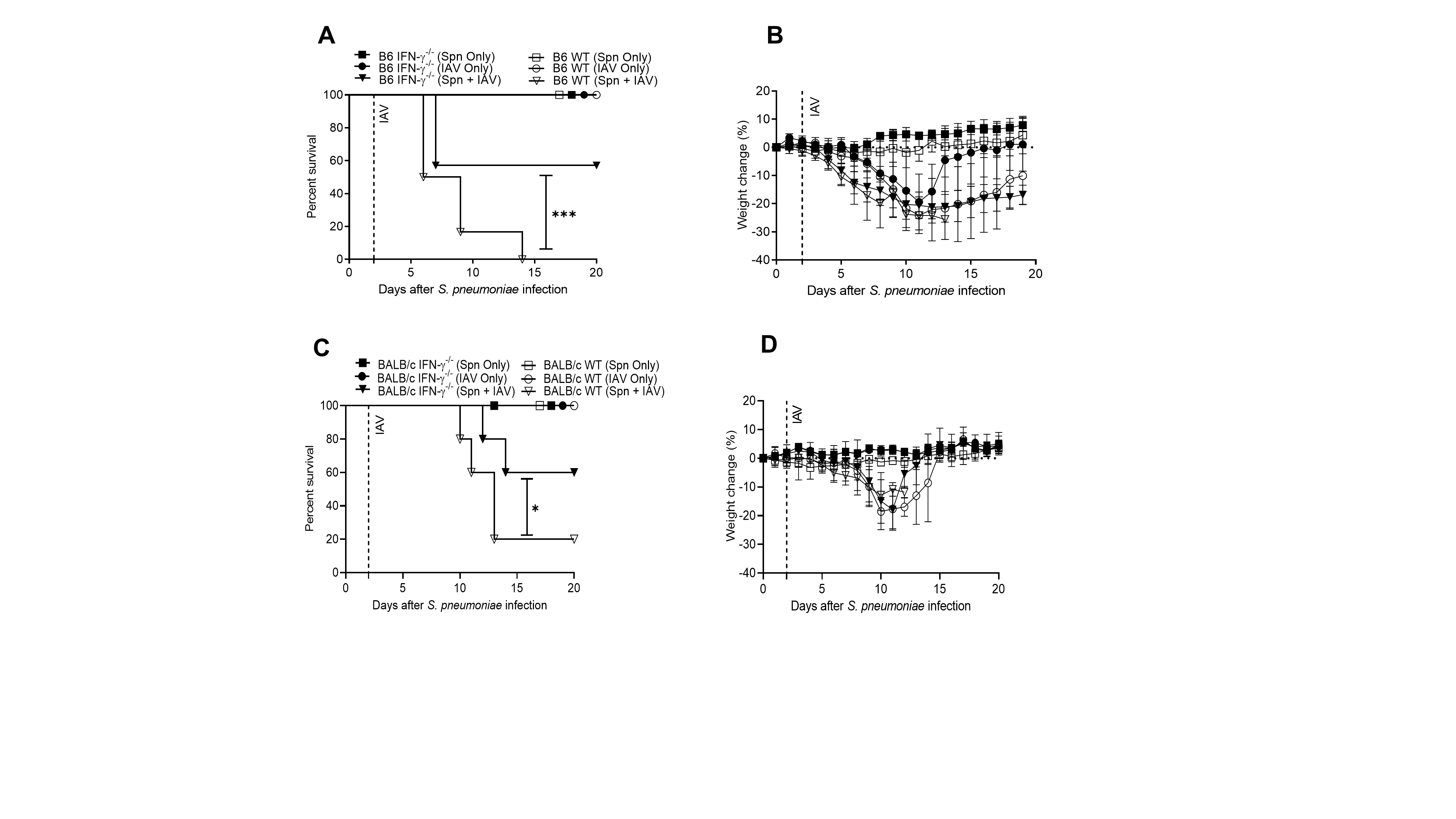

Supplement: S6 Fig — (A, B), C57BL/6 WT and IFN-γ-/- mice were infected on Day 0 with Spn alone, on Day 2 with IAV alone, or co-infected on the indicated days and monitored for (A) survival and (B) weight loss. 6–7 co-infected mice/group; 4 singly-infected mice/group. (C, D), BALB/c WT and IFN-γ-/- mice were infected on Day 0 with Spn alone, on Day 2 with IAV alone, or co-infected on the indicated days and monitored for (C) survival and (D) weight loss. 5 co-infected mice/group; 3 singly-infected mice/group. Survival data were analyzed by log-rank Mantel-Cox test. *P<0.05; **P<0.01; ***P<0.001. (TIF) [file ppat.1009405.s006.TIF]

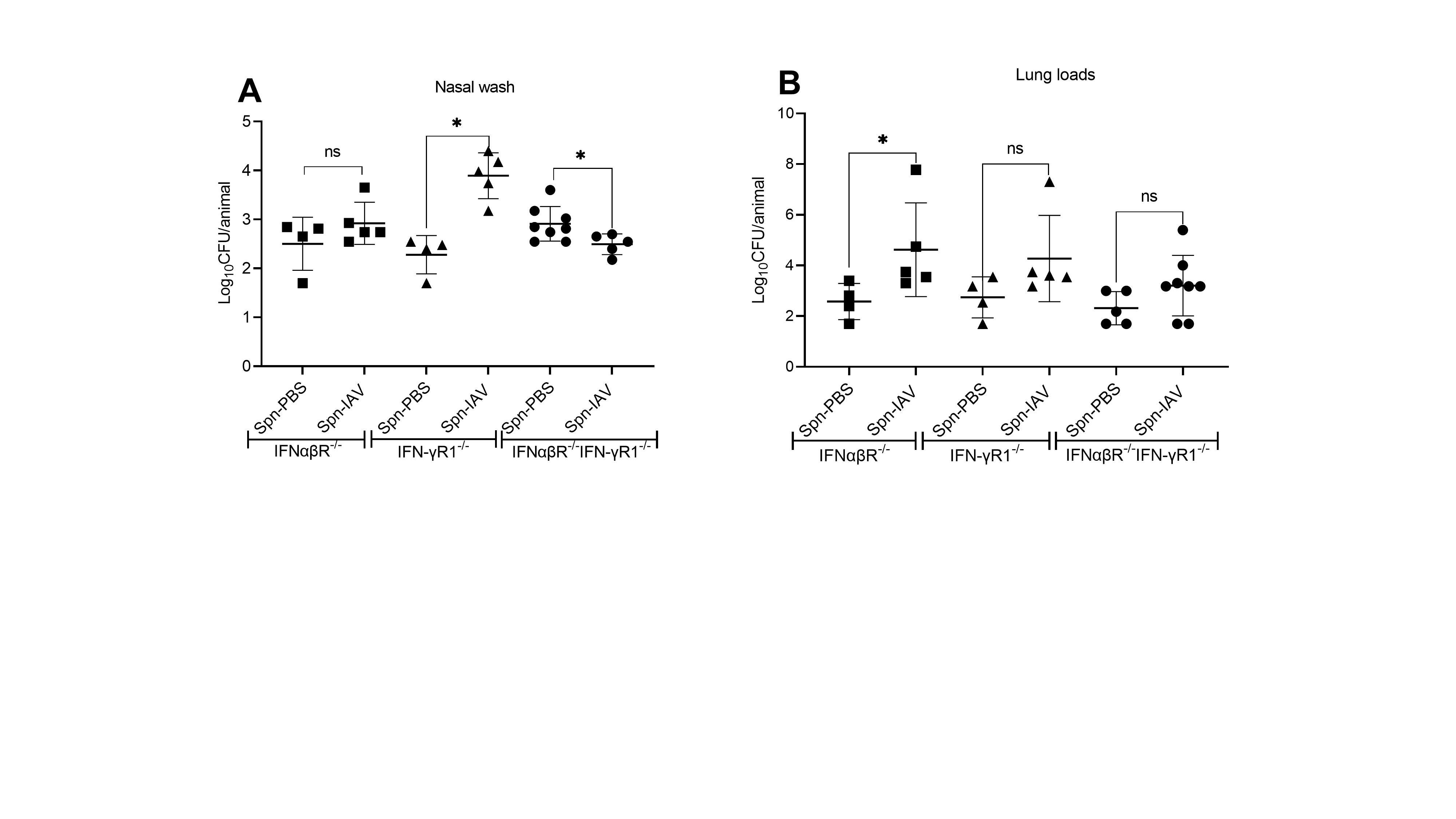

Supplement: S7 Fig — Nasal washes (A) and lung tissues (B) were analyzed on Day 7 after infection. Statistical analyses were performed by two-way ANOVA. *P<0.05; ***P<0.001; ****P<0.0001; ns = not significant. (TIF) [file ppat.1009405.s007.TIF]

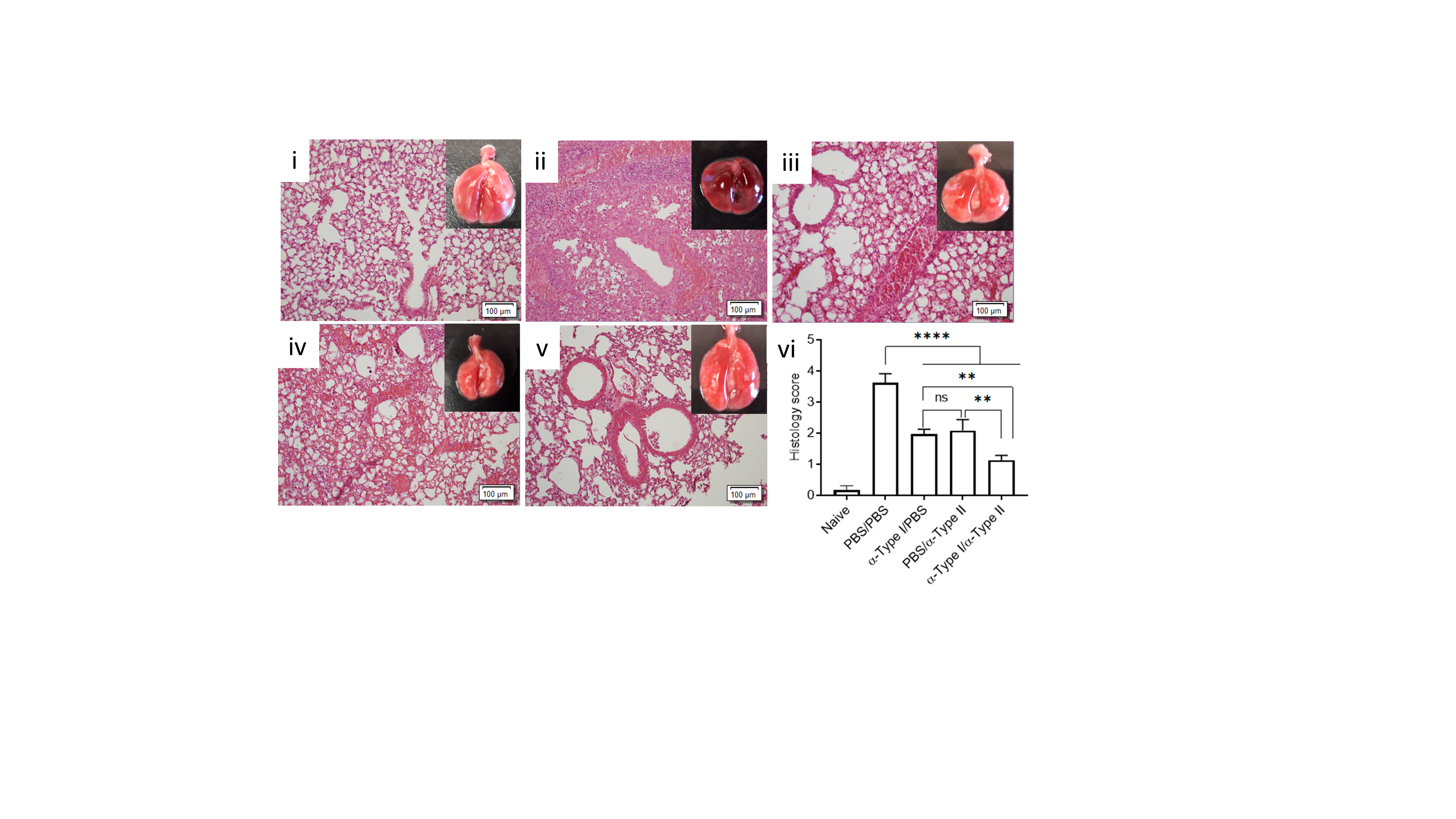

Supplement: S8 Fig — (i) naïve mouse; (ii) mouse treated with PBS and analyzed on Day 5 after co-infection; (iii) mouse treated with α-Type-I IFN/PBS and analyzed on Day 13 after co-infection; (iv) mouse treated with PBS/α-Type-II IFN and analyzed on Day 13 after co-infection; and (v) mouse treated with α-Type-I IFN/α-Type-II IFN and analyzed on Day 13 after co-infection. 20X magnification, scale = 100μm. Also shown is the representative gross pathology of the whole lungs; and (vi) Pathology scoring for 4 mice/group. Statistical analyses were performed by two-way ANOVA. **P<0.01, ****P<0.0001; ns = not significant. (TIF) [file ppat.1009405.s008.TIF]
